# Structure and Expression Analysis of *PtrSUS*, *PtrINV*, *PtrHXK*, *PtrPGM*, and *PtrUGP* Gene Families in *Populus trichocarpa* Torr. and Gray

**DOI:** 10.3390/ijms242417277

**Published:** 2023-12-08

**Authors:** Shuang Zhang, Wenjie Wang, Ruhui Chang, Jiajie Yu, Junxin Yan, Wenxi Yu, Chunming Li, Zhiru Xu

**Affiliations:** 1 College of Life Science, Northeast Forestry University, Harbin 150040, China; shuangzhang_1994@126.com (S.Z.); wangwj977@163.com (W.W.); crh1107@126.com (R.C.); 2 State Key Laboratory of Tree Genetics and Breeding, Northeast Forestry University, Harbin 150040, China; 13514554189@163.com; 3 College of Landscape Architecture, Northeast Forestry University, Harbin 150040, China; yanjunxin@163.com; 4 Heilongjiang Forestry Academy of Science, Harbin 150081, China; lkyyuwenxi@126.com

**Keywords:** poplar, sucrose synthase, invertase, hexokinase, phosphoglucomutase, UDP-glucose pyrophosphorylase, structural characteristic, gene expression, structural carbon

## Abstract

Exogenous nitrogen and carbon can affect plant cell walls, which are composed of structural carbon. Sucrose synthase (SUS), invertase (INV), hexokinase (HXK), phosphoglucomutase (PGM), and UDP-glucose pyrophosphorylase (UGP) are the key enzymes of sucrose metabolism involved in cell wall synthesis. To understand whether these genes are regulated by carbon and nitrogen to participate in structural carbon biosynthesis, we performed genome-wide identification, analyzed their expression patterns under different carbon and nitrogen treatments, and conducted preliminary functional verification. Different concentrations of nitrogen and carbon were applied to poplar (*Populus trichocarpa* Torr. and Gray), which caused changes in cellulose, lignin, and hemicellulose contents. In poplar, 6 *SUS*s, 20 *INV*s, 6 *HXK*s, 4 *PGM*s, and 2 *UGP*s were identified. Moreover, the physicochemical properties, collinearity, and tissue specificity were analyzed. The correlation analysis showed that the expression levels of *PtrSUS3*/*5*, *PtrNINV1*/*2*/*3*/*5*/*12*, *PtrCWINV3*, *PtrVINV2*, *PtrHXK5*/*6*, *PtrPGM1*/*2*, and *PtrUGP1* were positively correlated with the cellulose content. Meanwhile, the knockout of *PtrNINV12* significantly reduced the cellulose content. This study could lay the foundation for revealing the functions of *SUS*s, *INV*s, *HXK*s, *PGMs*, and *UGP*s, which affected structural carbon synthesis regulated by nitrogen and carbon, proving that *PtrNINV12* is involved in cell wall synthesis.

## 1. Introduction

All plant cells are surrounded by a cell wall, which determines the direction toward which the cells grow and protects plant cells from environmental damage. Plant cell walls are mainly composed of polysaccharides (cellulose, hemicellulose, and pectin), which are the largest sinks of carbon (C) fixation [1]. Sucrose transport from the photosynthetic tissue to the cell wall requires a highly complex process involving multiple enzymes [2]. Most of the sucrose produced by photosynthesis is transported to sink tissues through the phloem, and the metabolism of sucrose in sink tissues depends on two unloading pathways: symplastic and apoplastic pathways [3]. Symplastic unloading is usually the most important pathway by which sucrose enters sink-tissue cells directly through plasmodesmata. In sink cells, sucrose can be reversibly degraded by Sucrose synthase (SUS) to UDP-glucose (UDP-Glc) and fructose (Fru), and it can be irreversibly converted to glucose (Glc) and Fru by invertase (INV). Glc is converted to glucose 6-phosphate (Glc6P) by hexokinase (HXK), and Glc6P is catalyzed to glucose 1-phosphate (Glc1P) by phosphoglucomutase (PGM). And as a substrate of the cellulose synthase complex (CSC), UDP-Glc can be produced by Glc1P under the catalysis of UDP-glucose pyrophosphorylase (UGP) and be directly used to synthesize cellulose. It can also be further converted to other NDP-sugars for the synthesis of hemicellulose and pectin [1,4].

In plants, SUS is encoded by a small family of genes, and multiple *SUS* genes have been identified in many plants. For example, 15, 14, 7, 6, 6, 14, and 6 *SUS*s have been identified in *Populus trichocarpa* Torr. and Gray [5], *Nicotiana tabacum* L. [6], *Gossypium arboreum* Linn. [7], *Arabidopsis thaliana* (L.) Heynh. [8], *Oryza sativa* L. [9], *Brassica juncea* (L.) Czern. [9], and *Hevea brasiliensis* (Willd. ex A. Juss.) Muell. Arg. [10], respectively. And these *SUS*s have the domains of SUS and glucose transferase. The expression pattern of *SUS*s varies in different tissues. However, the six *AtSUS*s have completely different spatiotemporal expression patterns in *A. thaliana* [8]. In *O. sativa*, *OsSUS1* is mainly expressed in elongated tissues, such as internodes [11]. Previous studies have shown that 15 *PtSUS*s are differentially expressed in the roots, stems, and leaves in *P. tomentosa* (Carr.) [5]. In *G. arboreum*, the other five homologous genes, except for *GaSus2* and *GaSus7*, are differentially expressed in the stems and fibers [7]. SUS plays an important role in the normal growth and development of plants. For example, *PvSUS1* overexpression increases plant height and biomass in *Panicum virgatum* L. [12]. In *G. arboreum*, the heterologous expression of *Solanum tuberosum* L. *SUS* promotes leaf growth and fiber elongation [13]. The levels of starch and ADP-glucose in mature seeds of transgenic *Zea mays* L. expressing *StSUS4*, which encodes an *S. tuberosum* SuSy isoform, were higher than those in mature seeds of wild-type (WT) *Z. mays* [14]. The heterologous expression of *PsnSuSy2* (*P. simonii* Carrière × *P. nigra* L.) increased the cell wall thickness and plant height in *N. tabacum* [15]. The inhibition of the SUS activity in *G. arboreum* and *P. tremula* L. × *tremuloides* Michx. resulted in delayed fiber initiation [16] and significantly reduced the wood density [17], respectively. However, the inhibition of the AtSUS activity in *A. thaliana* had no significant effect on plant growth and development [18].

In plants, INV is a multigene family, which is divided into two subfamilies (acidic invertases and neutral/alkaline invertases) according to the optimal pH, and acidic invertases are further divided into cell wall invertase (CWINV) and vacuolar invertase (VINV) according to their subcellular localization [19,20,21]. However, the neutral/alkaline invertase (NINV) subfamily is usually localized in the cytoplasm, mitochondria, and chloroplasts [22]. To date, 17, 19, and 24 *INV* genes have been identified in *Arabidopsis* [22], *O. sativa* [22], and *P. trichocarpa* [23], respectively. In addition, acidic invertases have 13 conserved motifs, and NINVs have 12 conserved motifs [22,23]. The expression patterns of plant *INV*s are complex. For example, in *P. trichocarpa*, *PtCWINV3* is highly expressed in leaves, but *PtCWINV5* is only expressed in roots and mature leaves. *PtVINV2* is highly expressed in stems and nodes, as well as *PtVINV3* in young leaves. The expression levels of *PtNINV8*, 9, and *12* are the highest in stems and the lowest in leaves [23]. In *A. thaliana*, *AtcwINV1* is expressed in stems, leaves, and roots, while *AtcwINV2* is expressed specifically in flowers [21]. In *O. sativa*, all the cell wall invertase genes, except for *OsCIN9*, are expressed in leaves and stems. *OsVIN1* is highly expressed in all the tissues, while *OsVIN2* is highly expressed in mature leaves, panicles, and roots [22]. Plant INVs are closely related to cellulose synthesis. For example, the expressions of yeast extracellular invertase (*AI*) and yeast cytoplasmic invertase (*CI*) in *N. tabacum* increased the cellulose content [24]. In *G. arboretum*, the overexpression of *GhVINV1* significantly increased the cellulose content, and the inhibition of *GhVINV1* expression resulted in a significant decrease in the cellulose content [25]. The main reason for the decrease in the cellulose content was the inhibition of fiber initiation [26]. In *A. thaliana*, the mutant *cinv1cinv2* showed a loss of anisotropic growth, decreased cellulose content, and abnormal CSC arrangement [27]. The growth of the *ljcinv1* mutant was stunted compared with that of WT in *Lotus corniculatus* Linn. [28]. In *P. tremula* × *tremuloides*, inhibition of *PtCINV12* expression led to decreased cellulose content [29].

The *HXK* gene family has been analyzed using biochemical, genetic, and/or bioinformatics methods in many plant species, including *Arabidopsis* [30], *Solanum lycopersicum* L. [31], *N. tabacum* [32], *O. sativa* [33], *Phyllostachys edulis* (Carrière) J. Houz. [34], *Z.mays* [35], and *Pyrus bretschneideri* Rehder [36]. In *P. edulis*, 9 of the 12 *PeHXKs* genes (all except *PeHXK1*, *PeHXK4*, and *PeHXK10*) were found to be highly expressed in stems [34]. In plants, HXK is a multifunctional protein that functions as a kinase to phosphorylate enzymes, and as a sugar sensor. This protein can inhibit the expression of some photosynthetic genes, as well as regulating cell metabolism and some sugar-related signaling pathways [34]. The heterologous expression of *AtHXK1* in *Lycopersicon esculentum* var. *cerasiforme* A. Gray resulted in reduced photosynthesis and slow growth, and ultimately induced senescence [37]. The heterologous expression of *PbHXK1* in *L. esculentum* affected the sugar content and plant growth [36]. In *N. tabacum*, virus-induced silencing of *NbHXK1* led to cell death [38]. In *O. sativa*, overexpression of *OsHXK5* and *OsHXK6* resulted in growth retardation [39].

In plants, the PGM family can be divided into plastidic PGM (pPGM) and cytosolic PGM (cPGM) subtypes [40,41]. There are many forms of cPGM in plants, but only one form of pPGM. For example, there are two cPGMs and one pPGM in *N. tabacum* [42] and *Arabidopsi*s [43], but only one cPGM and one pPGM in *Spinacia oleracea* L. [44] and *S. tuberosum* [45]. In plant cells, cPGM is involved in photosynthesis, respiration, and cell wall synthesis, and plays an important role in sucrose metabolism [43,46]. Transgenic *S. tuberosum* showed decreased plant growth and tuber yield, decreased cPGM activity (by 61–85%), and changes in the rate of Glc metabolism [45,47]. The lack of cPGM activity in *A. thaliana* led to decreased rosette fresh weight, root length, and seed yield, as well as altered carbohydrate distribution [48]. The *pgm2/3* mutant showed changes in cell wall composition and decreased root length [48]. In plants, starch synthesis requires the participation of pPGM, ADP-glucose pyrophosphorylase, and starch synthase [49,50]. In studies on Glc metabolism in *L. corniculatus*, the leaves could not accumulate starch and could not be positively stained with iodine without pPGM [51]. When *pPGM* was knocked-down using an antisense method in *S. tuberosum*, the starch content in tubers was significantly decreased, but the sucrose content was significantly elevated [52]. The loss of both *cPGM* and *pPGM* in *A. thaliana* seriously affected plant growth and development, leading to decreased plant height and premature death [48].

UGP is an important enzyme in the synthesis of UDP-Glc, which is a precursor for the synthesis of cellulose, hemicellulose, and other carbohydrate components of the cell wall. Genes encoding UGPs have been cloned from *A. thaliana* [53], *S. tuberosum* [54], and *O. sativa* [55], and their expression patterns have been determined in a variety of plants. For example, *UGP* was found to be expressed in all tissues of *Musa acuminata* Colla, and its expression was up-regulated by exogenous sucrose and Fru [56]. In *S. tuberosum*, *UGP* was expressed in all tissues and its expression was up-regulated by exogenous sucrose [57]. In *S. tuberosum* tubers, decreased UGP activity resulted in a significant decrease in sucrose content [58,59]. Overexpression of bacterial *UGP* in *N. tabacum* resulted in increased plant height [60]. In *O. sativa*, *UGP1* was shown to be essential for plant growth and reproduction [61,62]. In the *Arabidopsis atugp1atugp2* mutant, genes encoding enzymes involved in the cellulose and callose synthesis pathways were down-regulated [53].

Nitrogen (N) and C are essential for the basic biological processes and normal growth of plants. In fact, N and C metabolism often interact with each other [63,64,65]. Application of exogenous N affects cell wall thickness in poplar [66,67]. The cell wall is the largest C pool in plants, and its thickness is regulated by the C supply [68,69]. It has been predicted that the dynamic balance between C and N metabolism will be significantly affected by global climate change and low N availability in soil [67,70,71]. Previous studies have shown that the production, flow, and decomposition of sucrose in poplar are affected by the CO_2_ concentration to different degrees [69]. Sucrose is the main sugar source for cell wall synthesis [4], but the mechanisms underlying the responses of enzymes involved in sucrose metabolization to N and C remain unclear. A previous study proposed that the properties of cellulose microfibers could be changed by altering the substrate supplied for cellulose biosynthesis [29] The secondary growth of woody plants exhibits typically compared to herbaceous plants, which results in a higher ratio of structural C to total biomass. Compared with *Arabidopsis*, *P. trichocarpa* has more protein-coding genes in its genome, with an average of 1.4 to 1.6 putative *P. trichocarpa* homologs for each *Arabidopsis* gene. Notably, there are overrepresented exceptions in *P. trichocarpa*, particularly for genes associated with lignocellulosic cell wall biosynthesis. For instance, *KOR*, *SuSY*, *COBRA*, and *FRA2* genes occur in duplicate pairs in *Populus*, but are single-copy genes in *Arabidopsis* [72]. In the transition from primary growth to secondary growth in *P. tremula* × *P. alba* (clone INRA 717 1B-4), there are changes in the expression patterns of certain genes related to secondary cell wall synthesis, such as *VINV*, *GS*, *CYP450*, *CAD*, *4CL*, and *UGT* [73]. These findings might suggest that the evolution of similar genes contributes to the advanced developmental characteristics of woody plants compared to herbaceous plants. In this study, we selected *P. trichocarpa* as the experimental material, because it is a model woody plant with typical secondary growth characteristics and a fully sequenced genome [72]. *P. trichocarpa* plants were treated with different concentrations of N and C, which eventually led to changes in the structural C content (lignin, cellulose, and hemicellulose). A genome-wide analysis of five functional gene families (*SUS*, *INV*, *HXK*, *PGM*, and *UGP*) associated with sucrose metabolism in *P. trichocarpa* was conducted using bioinformatics methods. In addition, real-time quantitative PCR (qRT-PCR) was used to study the expression patterns of the five gene family members, and correlation analysis of genes with structural C content under different N and C treatments. To confirm the relationship between certain genes and structural C content, *PtrNINV12* was selected for further analyses. The knockout of *PtrNINV12* significantly reduced the cellulose content, indicating that *PtrNINV12* is indeed involved in the synthesis of structural C. Our study not only offers a comprehensive genome-wide analysis of gene family members associated with sucrose metabolism, but also suggests their potential involvement in the synthesis of cell walls under the treatments of N and C.

## 2. Results

### 2.1. Changes in Plant Growth and Development in Response to Exogenous C and N Treatments

**Figure 1:**
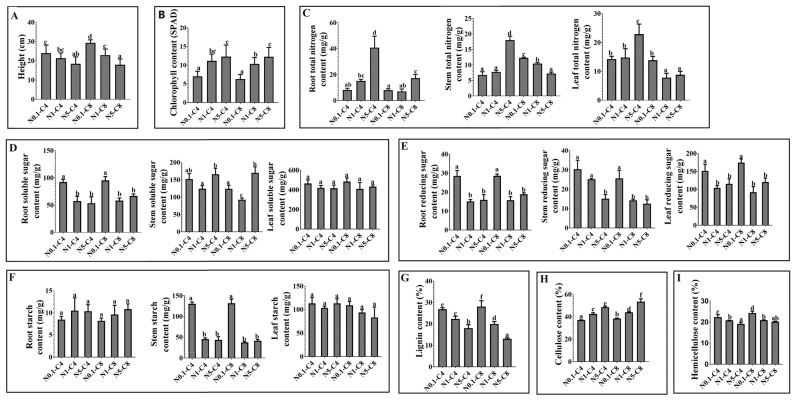
Plant height and contents of chlorophyll, total N, soluble sugar, reducing sugar, starch, lignin, cellulose, and hemicellulose under different treatments. (**A**) Plant height; figure shows difference in plant height between before and after treatments. (**B**) Chlorophyll content. (**C**) Total N content in roots, stems, and leaves. (**D**) Soluble sugars content in roots, stems, and leaves. (**E**) Reducing sugar content in roots, stems, and leaves. (**F**) Starch content in roots, stems, and leaves. (**G**) Lignin content in stems. (**H**) Cellulose content in stems. (**I**) Hemicellulose content in stems. Same lowercase letters indicate insignificant differences between the lines tested using Duncan’s multiple range test with significance level of *p* < 0.05.

To understand the effects of exogenous N and C on poplar growth, plant height and chlorophyll content were measured. As shown in Figure 1A,B, chlorophyll content increased and plant height decreased with increasing N concentrations under the same C concentration. Under the same N conditions, plant height was greater in the C8 treatment than in the C4 treatment, but the chlorophyll content was similar in the C8 and C4 treatments. To explore the effects of exogenous N and C on the N content of plants, the changes in total N contents were determined. Compared with the control, the total N content elevated with the increased N concentrations in roots, stems, and leaves under C4 conditions. Under C8 conditions, the trend remained the same with that under C4 condition in roots, but was opposite in leaves and stems (Figure 1C).

To further explore the effects of exogenous N and C on the C content of plants, the changes in the contents of soluble sugar, reducing sugar, starch, lignin, cellulose, and hemicellulose were determined. As shown in Figure 1D, under the same C conditions, the soluble sugar content in roots was highest in the N0.1 treatment, but similar in the N1 and N5 treatments; while the soluble sugar content in stems was highest in the N5 treatment, followed by the N0.1 treatment, and lowest in the N1 treatment. In the leaves, the trend in the change in soluble sugar content was similar to that observed in the roots, but was not significant. Under the same N conditions, the soluble sugar contents in roots and leaves were similar in the C4 and C8 treatments, but in stems, the soluble sugar content was higher in the C4 treatment than in the C8 treatment. The effects of exogenous C and N treatments on the reducing sugar content is shown in Figure 1E. The change trend of reducing sugar contents in roots and leaves was similar to that of soluble sugar content in roots. Under C4 conditions, the reducing sugar content in stems decreased with increasing N concentrations. This trend was also observed under C8 conditions, but the reducing sugar content was lower in the C8 treatments than in the C4 treatments under the same N conditions. The starch contents in roots and leaves were not significantly affected by C and N treatments, but the trend in the change in starch content in roots was opposite to that of the soluble sugar content in the roots (Figure 1F). The starch contents in stems were highest in the N0.1-C4 and N0.1-C8 treatments (Figure 1F). Lignin, cellulose, and hemicellulose are structural C that are concentrated in the stems, so their contents in stems were determined. As shown in Figure 1G–I, under C4 conditions, the lignin and hemicellulose contents decreased significantly as the N concentration increased, while the cellulose content increased significantly. The same trends were observed under C8 conditions, but the magnitude of the changes was larger. The data for all these indexes are shown in Table S1.

Exogenous application of N and C not only affected the contents of C-related and N-related metabolites, but also the contents of cellulose and hemicellulose, the final form of stored C in the cell wall. Therefore, *SUS*, *INV*, *HXK*, *PGM*, and *UGP*, which are involved in cell wall C metabolism, were genome-wide analyzed by bioinformatics to investigate the relevance between their gene expressions and the above indexes. 

### 2.2. Analysis of the PtrSUS, PtrINV, PtrHXK, PtrPGM, and PtrUGP Family Members in P. trichocarpa

To identify and analyze the PtrSUS, PtrINV, PtrHXK, PtrPGM, and PtrUGP family in *P. trichocarpa*, AtSUSs, AtINVs, AtHXKs, AtPGMs, and AtUGPs were used as probe sequences. Finally, 6 *SUS*s, 20 *INV*s, 6 *HXK*s, 4 *PGM*s, and 2 *UGP*s were identified in *P. trichocarpa* genome. To further understand the basic characteristics of these genes, the physiochemical properties of the putative proteins were investigated (Table S2). The results were as follows: the number of amino acids in PtrSUSs, PtrINVs, PtrHXKs, PtrPGMs, and PtrUGPs were in the ranges of 804–922, 556–663, 491–509, 583–638, and 470; and the putative proteins had isoelectric points of 5.93–8.04, 4.98–8.6, 5.63–6.54, 5.31–5.9, and 5.51–5.78, respectively. The grand average of hydropathy (GRAVY) values of PtrSUSs, PtrINVs, PtrHXKs (except PtrHXK2, 5, and 6), PtrPGMs, and PtrUGPs were negative, indicating that they were hydrophilic proteins. The subcellular localization prediction analyses showed that PtrSUS1–3 were located in the cytosol, but PtrSUS5–6 were located in the chloroplast. It was predicted that PtrNINVs were mostly located in the cytosol and chloroplasts, whereas PtrCWINVs were located in the extracellular region and PtrVINVs were located in vacuoles. All PtrHXKs were predicted to localize in chloroplasts. It was predicted that PtrPGM1–2 were localized in the cytosol (cPGMs) and PtrPGM3–4 were localized in chloroplasts (pPGM). PtrUGP1–2 were predicted to localize in the cytosol.

To explore the conservation of PtrSUS, PtrINV, PtrHXK, PtrPGM, and PtrUGP family members, multiple sequence alignments were performed (Figure S1). The results revealed high conservation among the members of each family. In the PtrSUS family, except for PtrSUS5, all of the PtrSUSs had conserved serine residues in the N-terminal region of their amino acid sequences, and all PtrSUSs had conserved sucrose synthetase and glycosyl transferase domains, the characteristic domains of SUS proteins in plants. All PtrNINVs had 12 conserved domains, and PtrCWINVs and PtrVINVs had 13 conserved domains, respectively. All PtrHXKs contained four conserved domains, namely the phosphate 1, phosphate 2, sugar-binding, and adenosine domains. In addition, the PtrHXK proteins included four additional peptides (loop 1–4) as reported for HXKs in other species [74]. PtrPGM1–4 had the conserved region of the Glc ring-binding domain (C–G–E–E–S–F). The catalytic reaction center and metal-binding domain of PtrPGM1–2 consisted of the amino acid sequences T–A–S–H–N and D–G–D–A–D, respectively. The amino acid sequences of the reaction center site and metal-binding site of PtrPGM3–4 were S–A–S–H–N and D–G–D–G–D, respectively. PtrUGP1–2 had nucleotide-binding, glycosylation, and proteolytic cleavage regions. 

### 2.3. Gene Structure and Phylogenetic Analysis

**Figure 2:**
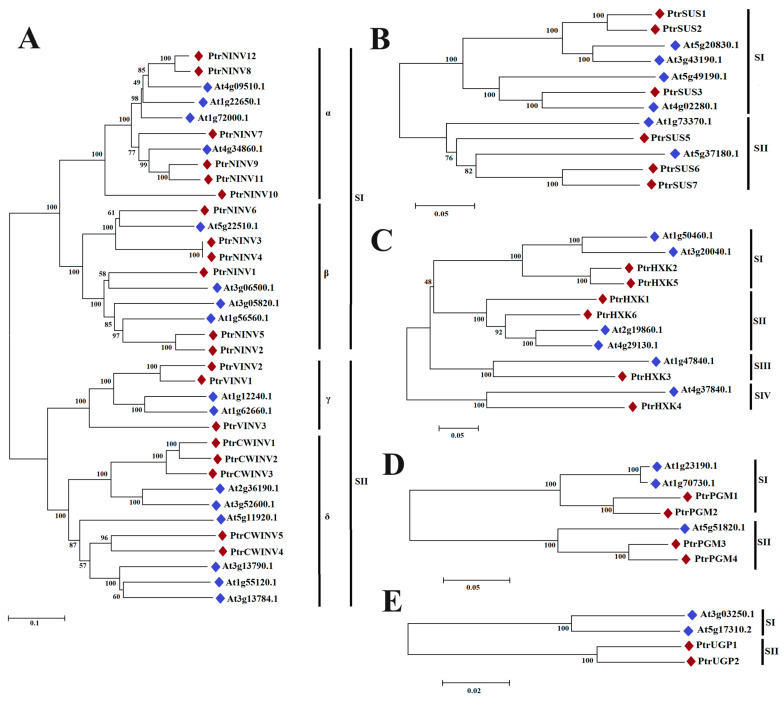
Phylogenetic analysis of INV (**A**), SUS (**B**), HXK (**C**), PGM (**D**) and UGP (**E**) from *P. trichocarpa* and *A. thaliana*. Phylogenetic trees were constructed using the neighbor-joining method. Red and blue diamonds represent proteins from *P. trichocarpa* and *A. thaliana*, respectively. α, β, γ, δ, SI, SII, SIII, and SIV represent different evolutionary branches (subgroups). Scale bar is shown below evolutionary trees.

To explore the conserved and evolutionary relationships of PtrSUS, PtrINV, PtrHXK, PtrPGM, and PtrUGP family members, evolutionary trees of corresponding genes in *P. trichocarpa* and *Arabidopsis* were constructed (Figure 2), and the distribution of introns/exons and conserved motifs in *P. trichocarpa* (Figure S2) were analyzed. The results showed that SUSs could be divided into two groups; SI and SII. PtrSUS1–3 belonged to the SI group, and PtrSUS5–7 belonged to the SII group. PtrSUS1/2 and PtrSUS3/5 contained 13 and 15 exons, respectively; and PtrSUS6 and 7 contained 12 and 14 exons, respectively. The conserved sucrose synthase domains and glycosyltransferase domains were distributed in 10 conserved motifs of PtrSUSs. The evolutionary tree constructed from PtrINV and AtINV sequences also revealed two subgroups, SI and SII. PtrNINV belonged to the SI group, and PtrCWINV and PtrVINV belonged to the SII group. The SI and SII groups were further divided into the α subgroup (PtrNINV7–12), β subgroup (PtrNINV1–6), γ subgroup (PtrVINV1–3), and δ subgroup (PtrCWINV1–5). PtrNINV1–6 in the β subgroup were encoded by genes containing six exons, while PtrNINV7–12 in α subgroup were encoded by genes containing four exons. PtrNINV1–12 had exactly the same 10 motifs, which included eight conserved domains (numbered 1–8) associated with the NINV subfamily. Except for PtrCWINV1/2 and PtrVINV1, members of the acid invertase subfamily were encoded by genes containing 7 exons and contained the same 10 motifs. The gene encoding PtrVINV1 had no introns, but encoded all 13 conserved domains (Figure S1) associated with the acid invertase subfamily. Notably, motif 7 was specific to PtrCWINV1 and motif 9 was specific to PtrCWINV2. The evolutionary tree constructed from HXK sequences was divided into four groups; SI, SII, SIII, and SIV. All PtrHXKs contained 9 exons and 10 conserved motifs except for PtrHXK3, which lacked motifs 7 and 8. The conserved domains were distributed in motif 2, motif 3, motif 4, motif 6, and motif 10. The PtrPGMs and AtPGMs were also divided into two groups; SI and SII. PtrPGM1/2 belonged to the SI group, and PtrPGM3/4 belonged to the SII group. The genes encoding PtrPGM1/2 had 18 exons and those encoding PtrPGM3/4 had 22 exons, which further proved that PtrPGM1/2 were cPGMs and PtrPGM3/4 pPGMs. The Glc-ring-binding, metal-binding, and catalytic reaction center domains corresponded to motif 1, motif 7, and motif 3, respectively. The UGPs were divided into two groups; SI and SII. PtrUGPs were highly conserved, all encoded by genes with 21 exons and containing exactly the same 10 motifs. In addition, the nucleotide-binding, glycosylation, and proteolytic cleavage domains corresponded to motif 7, motif 1, and motif 5, respectively. The sequences of the conserved motifs in the PtrSUS, PtrINV, PtrHXK, PtrPGM, and PtrUGP proteins are listed in Table S3.

### 2.4. Chromosome Distribution and Collinearity Analysis

**Figure 3:**
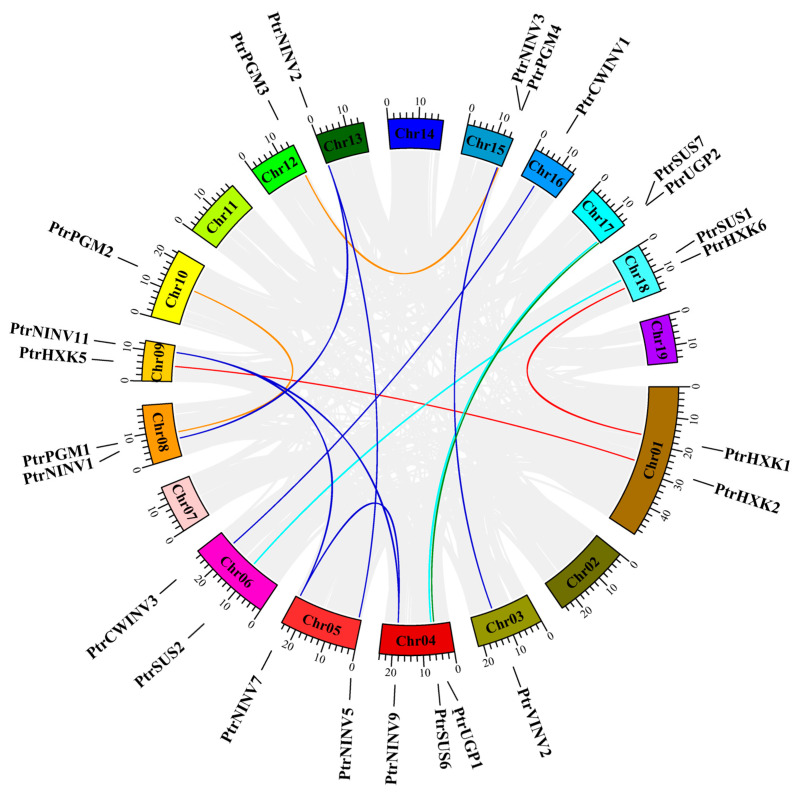
Schematic representations of segmental duplications of *PtrSUS*, *PtrINV*, *PtrHXK*, *PtrPGM*, and *PtrUGP* genes. Gray, cyan, blue, red, yellow, and green lines indicate all syntenic blocks between each chromosome in the poplar genome and duplicated *PtrSUS*, *PtrINV*, *PtrHXK*, *PtrPGM*, *PtrUGP* gene pairs, respectively. Ratio bars represent chromosome length (Mb), with gene names shown alongside. Chromosome number is shown below each chromosome. Different chromosomes are shown in different colors.

To further investigate the evolution of *PtrSUSs*, *PtrINVs*, *PtrHXKs*, *PtrPGMs*, and *PtrUGP*s, chromosome localization analysis (Figure S3) and collinearity analysis among gene family members (Figure 3) were carried out. The results showed that six *PtrSUS*s were located on six different chromosomes. *PtrSUS1/2* and *PtrSUS6/7* were located on Chr18/06 and Chr04/17, respectively, and showed fragment replication relationships. 20 *PtrINV*s were located on 12 different chromosomes, and *PtrCWINV1/2* and *PtrCWINV4/5* were identified as tandem replicates on Chr16 and Chr06, respectively. Fragment replication relationships were detected between *PtrNINV9* and *PtrNINV7/11*; and for *PtrNINV7/11*, *PtrNINV5/2*, *PtrNINV1/2*, *PtrVINV2/3*, and *PtrCWINV3/1* on Chr05/09, Chr05/13, Chr08/13, Chr03/15, and Chr06/16, respectively. The *PtrHXK* genes were distributed on five chromosomes. The fragment replication relationships detected for *PtrHXK* genes were as follows: *PtrHXK2* on Chr01 and *PtrHXK5* on Chr09, and *PtrHXK1* on Chr01 and *PtrHXK6* on Chr18. The *PtrPGM* genes were distributed on Chr08, 10, 12, and 15. Fragment replication relationships were detected for *PtrPGM1/2* on Chr08/10 and for *PtrPGM3/4* on Chr12/15. A fragment replication relationship between *PtrUGP1* and *2* was also detected.

**Figure 4:**
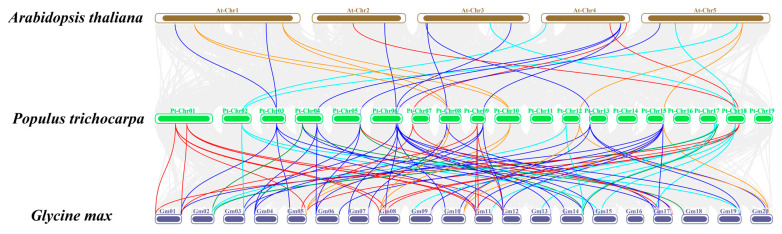
Synteny analysis of *SUS*, *INV*, *HXK*, *PGM*, and *UGP* genes among *P. trichocarpa*, *A. thaliana*, and *G. max*. Gray, cyan, blue, red, yellow, and green lines represent collinear blocks among *P. trichocarpa* and other plant genomes, as well as *PtrSUS*, *PtrINV*, *PtrHXK*, *PtrPGM*, and *PtrUGP* gene pairs. Ends of lines connect the chromosomes, with the chromosome number at the top and the species name on the left. Different colors distinguish chromosomes of *P. trichocarpa*, *A. thaliana*, and *G. max*.

To further investigate the evolutionary relationships of *SUSs*, *INVs*, *HXKs*, *PGMs*, and *UGPs* among different species, synteny analyses of these genes in *P. trichocarpa*, *A. thaliana*, and *Glycine max* (L.) Merr. were performed (Figure 4). The results showed that the *SUSs*, *INVs*, *HXKs*, *PGMs*, and *UGPs* of *P. trichocarpa* were highly homologous to those of *G. max*, with 12, 33, 20, 8, and 5 orthologous homologous gene pairs, respectively, between *P. trichocarpa* and *G. max*. Comparing *P. trichocarpa* and *Arabidopsis*, the number of homologous gene pairs of *SUSs*, *INVs*, *HXKs*, *PGMs*, and *UGPs* was 4, 10, 3, 6, and 0, respectively. The *SUS*, *INV*, *HXK*, and *UGP* gene families of *P. trichocarpa* had some members that were related to at least three pairs of homologous genes in corresponding families of *Arabidopsis* or *G. max*, such as *PtrSUS1*, *PtrSUS7*, *PtrCWINV3*, *PtrNINV2*, *PtrHXK5*, *PtrHXK6*, and *PtrUGP1*. Therefore, these genes may have played important roles in the evolution of *SUS*, *INV*, *HXK*, and *UGP* genes [75]. 

### 2.5. Secondary and Tertiary Structure Prediction

**Figure 5:**
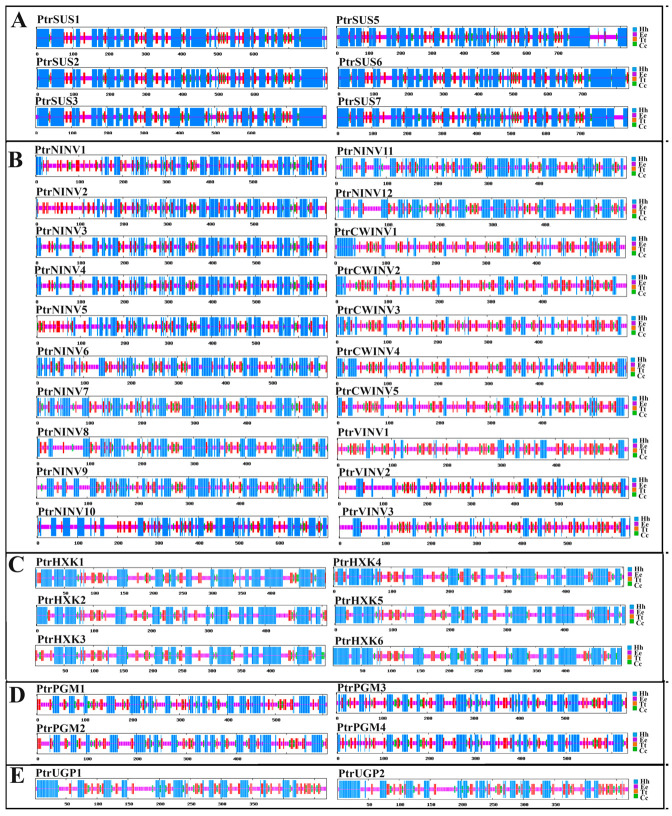
Secondary structures of PtrSUS (**A**), PtrINV (**B**), PtrHXK (**C**), PtrPGM (**D**), and PtrUGP (**E**) proteins. Secondary structure motifs of proteins are represented by different colors: blue, α-helix (Hh); purple, random coil (Cc); red, extended strand (Ee); green, β-turn (Tt).

**Figure 6:**
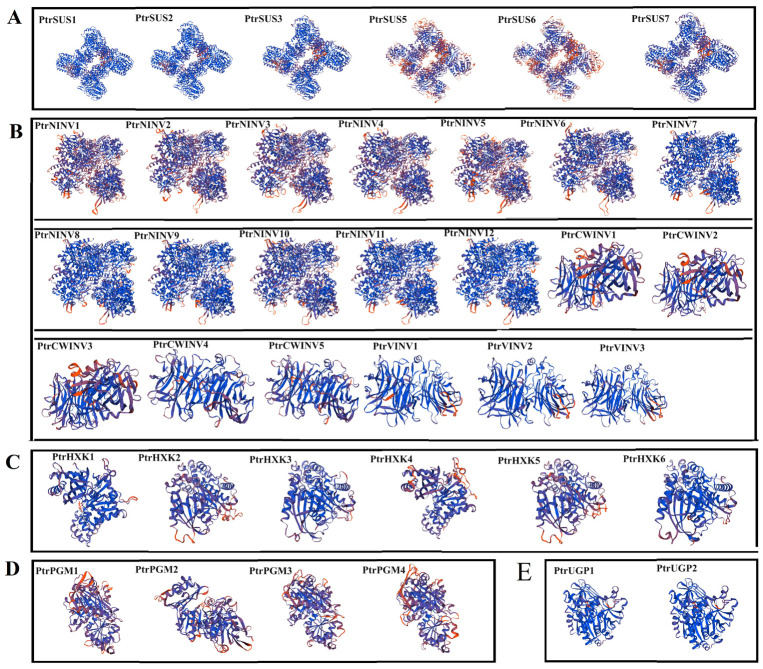
Tertiary structures of PtrSUS (**A**), PtrINV (**B**), PtrHXK (**C**), PtrPGM (**D**), and PtrUGP (**E**) proteins.

The structure and function of proteins are highly unified [76], so to further clarify the functions of the various proteins, their secondary and tertiary structures were predicted. Four secondary structure elements were identified in the PtrSUS, PtrINV, PtrHXK, PtrPGM, and PtrUGP proteins: α helixes (Hh), β turns (Tt), extended strands (Ee), and random coils (Cc). In PtrSUSs and PtrHXKs, Hh accounted for the largest proportion of amino acids, followed by Cc. In PtrPGMs, Cc accounted for the largest proportion of amino acids, followed by Hh. In PtrUGPs, Cc and Hh accounted for similar numbers of amino acids, followed by Ee. In PtrNINVs, Cc and Hh accounted for similar proportions of amino acids, whereas in PtrCWINVs and PtrVINVs, Cc accounted for the largest proportion of amino acids, followed by Ee (Figure 5, Table S4). The results of tertiary structure analyses showed that PtrSUSs had similar tertiary structures and functioned as a homotetramers. The tertiary structures of PtrNINVs were similar, and all of them functioned as homohexamers. In addition, PtrCWINV1–3 had the same tertiary structure, PtrCWINV4–5 and PtrVINV1–3 had the same tertiary structure, and all of them executed their functions in monomer form. A variety of tertiary structures were predicted for PtrHXKs, suggesting that various PtrHXKs had different functions. However, all the PtrHXKs were predicted to function in monomer form. PtrPGM1/3/4 had a similar tertiary structure that differed from that of PtrPGM2. The two PtrUGPs were highly conserved and had a similar tertiary structure, and both of them were predicted to function in monomer form (Figure 6, Table S5).

### 2.6. Tissue-Specific Expression Analysis

**Figure 7:**
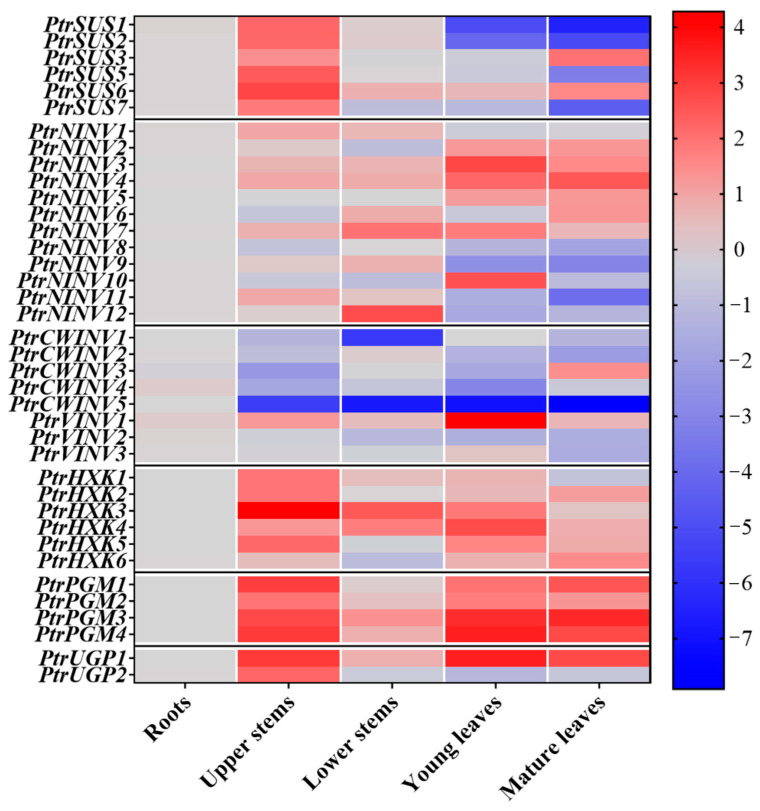
Transcript profiles of various genes in roots, upper stems, lower stems, mature leaves, and young leaves. Gene transcript levels of *PtrSUS*, *PtrINV*, *PtrHXK*, *PtrPGM*, and *PtrUGP* were calculated using the 2^−ΔΔCt^ method, and the relative transcript level of each gene is shown as log_2_ value. Scale bar is shown on the right side of the heatmap.

To explore the expression pattern of *PtrSUSs*, *PtrINVs*, *PtrHXKs*, *PtrPGMs*, and *PtrUGPs* in different tissues, the RNA of upper stems, lower stems, young leaves, mature leaves, and roots was extracted and then reversely transcribed into cDNA for qRT-PCR. As shown in Figure 7, among five tissues, the upper stems showed very high transcript levels of *PtrSUS*, *PtrHXK*, *PtrPGM*, and *PtrUGP*. Most members of the *PtrSUS* family (except *PtrSUS3* and *6*) showed lower transcript levels in young or mature leaves than in roots and lower stems. Most genes in the *PtrHXK* (except *PtrHXK1*), *PtrPGM*, and *PtrUGP* (except *PtrUGP2*) families showed higher transcript levels in young or mature leaves than in roots and lower stems. The transcript profiles of the *PtrINV* family genes in the five tissues were complex. In the *PtrNINV* subfamily, the transcript levels of *PtrNINV1*/*2*/*3*/*4*/*6*/*7*/*9*/*11*/*12* genes in upper stems or lower stems were higher than those in roots. The difference was that the expression levels of *PtrNINV1*/*9*/*11*/*12* in upper stems or lower stems were higher than those in young and mature leaves, but *PtrNINV2*/*3*/*4* showed the opposite expression pattern with *PtrNINV1*/*9*/*11*/*12*. *PtrCWINV3* was highly expressed in the mature leaves, whereas all other members of the *PtrCWINV* subfamily were highly expressed in the roots. The transcript levels of *PtrCWINV2*/*3*/4 in lower stems were similar to those in the roots, and were higher in roots and lower stems than in young leaves. In the *PtrVINV* subfamily, *PtrVINV1/3* showed higher transcript levels in young leaves than in upper stems, lower stems, young leaves, and mature leaves. The transcript levels of *PtrVINV2* were higher in the roots and upper stems than in the lower stems, young leaves, and mature leaves. The related qRT-PCR data are shown in Table S6.

### 2.7. Effects of Different Treatments on Expression Patterns of PtrSUSs, PtrINVs, PtrHXKs, PtrPGMs, and PtrUGPs

**Figure 8:**
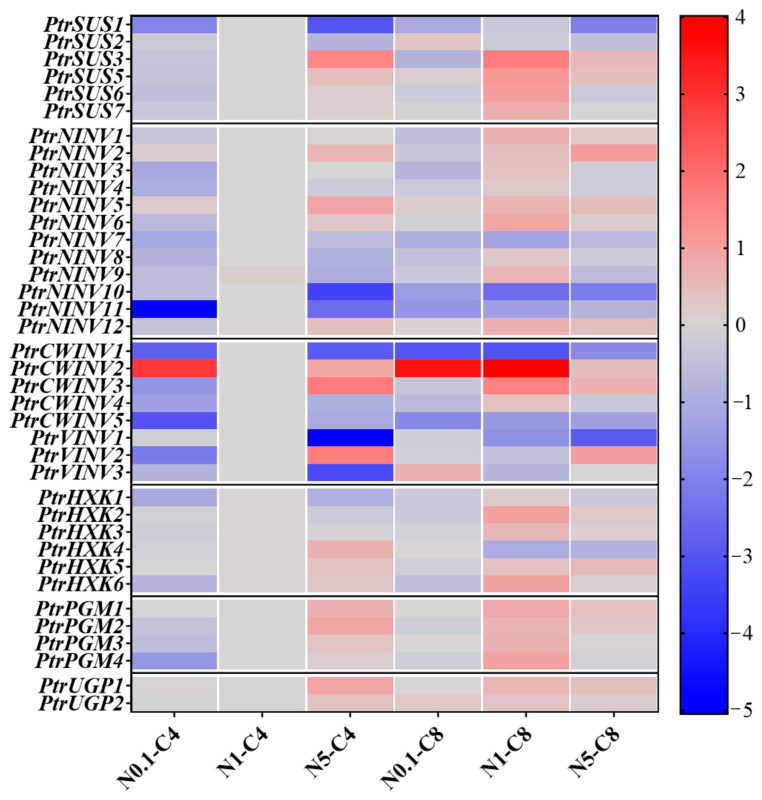
Transcript profiles of *PtrSUSs*, *PtrINVs*, *PtrHXKs*, *PtrPGMs*, and *PtrUGPs* in different treatments, as determined by qRT-PCR. Relative transcript levels were calculated using the 2^−ΔΔCT^ method, and are shown using log_2_ values (sample/control) of each gene under different treatments. In heat maps, the scale bar is shown on the right, and different cell colors indicate whether each gene was up-regulated or down-regulated in each treatment compared with the control. Significant differences in gene transcript levels were determined using Duncan’s multiple range test (*p* < 0.05). Gene transcript levels in different treatments are summarized in Table S6.

To understand the response of *PtrSUSs*, *PtrINVs*, *PtrHXKs*, *PtrPGMs*, and *PtrUGPs* to N and C conditions (N0.1-C4, N1-C4, N5-C4, N0.1-C8, N1-C8, and N5-C8), the relative expression levels of genes were analyzed using qRT-PCR. As shown in Figure 8, under C4 conditions, the transcript levels of most genes increased with increasing N concentrations. For example, the transcript levels of *PtrSUS3*/*5*/*6*, *PtrNINV6/12*, *PtrCWINV3*, *PtrVINV2*, *PtrHXK6*, *PtrPGM2*/*3*, and *PtrUGP1/2* significantly increased with increasing N concentrations. However, for some other genes (*PtrSUS1*/*2*, *PtrNINV7*/*8*/*9*/*11*, *PtrCWINV1*/*4*/*5*, *PtrVINV3*, and *PtrHXK1*), their transcript levels were significantly lower in N0.1-C4 and N5-C4 treatments than in the control. Under N1 conditions, the transcript levels of most genes were higher in the C8 treatment than in the C4 treatment, but a few genes showed decreased transcript levels with higher C concentrations. For example, the transcript levels of *PtrSUS3*/*5*/*6/7*, *PtrNINV1*/*3*/*4*/*5*/*6*/*8*/*9*/*12*, *PtrCWINV2*/*3*, *PtrHXK3*/*5*, *PtrPGM1*/*2*/*3*/*4*, and *PtrUGP1/2* were significantly higher in the N1-C8 treatment than the N1-C4 treatment; but those of *PtrSUS1*/*2*, *PtrNINV7*/*10*, *PtrCWINV1*/*5*, *PtrVINV1*/*2*/*3*, and *PtrHXK4*/*6* were significantly lower in the N1-C8 treatment than the N1-C4 treatment. Under C8 conditions, most genes had low transcript levels in the N0.1 treatment, but higher transcript levels in the N5 treatment. For example, in the N0.1-C8 treatment, the transcript levels of *PtrSUS1*/*3*/*6*, *PtrNINV1*/*3*/*7*/*8*/*9*/*10*/*11*, *PtrCWINV1*/*3*/*4*/*5*, *PtrVINV2*, and *PtrHXK1/6* were significantly lower than those in the control, whereas in the N5-C8 treatment, the transcript levels of *PtrSUS3*/*5*, *PtrNINV2*/*5*/*6*/*12*, *PtrCWINV2*/*3*, *PtrVINV2*, *PtrHXK5*, *PtrPGM1*/*2*, and *PtrUGP1/2* were significantly higher than in the control. Interestingly, in stems, some genes showed consistent responses to different treatments. The transcript levels of *PtrSUS1*, *PtrNINV7*, *PtrCWINV1*/*5*, and *PtrHXK1* were significantly lower in all treatments than in the control, while that of *PtrCWINV2* was significantly higher in all treatments than in the control.

### 2.8. Correlation Analysis between Expression Levels of PtrSUSs, PtrINVs, PtrHXKs, PtrPGMs, and PtrUGPs and Physiological Characteristics under Different Treatments

**Figure 9:**
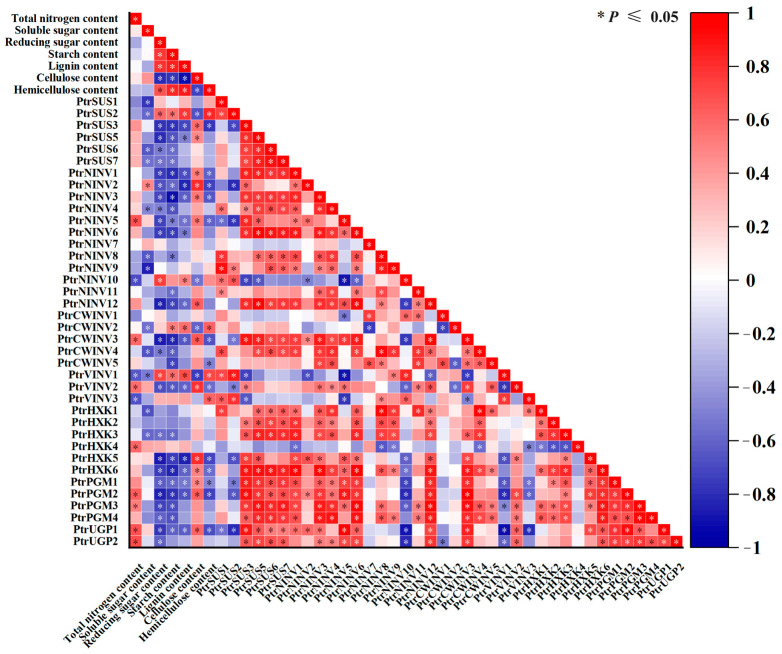
Correlation analyses between contents of total N, soluble sugar, reducing sugar, starch, lignin, cellulose, and hemicellulose and transcript levels of *PtrSUS*, *PtrINV*, *PtrHXK*, *PtrPGM*, and *PtrUGP* genes under different treatments. In the maps, scale bar is shown at right, and the different color of the cells indicate the degree of correlation. Asterisk (*) indicates significant correlation.

To verify our hypothesis that changes in gene transcript levels were related to changes in the contents of structural C, correlation analysis was conducted between physiological characteristics and expression patterns of each family member under the N and C treatments. As shown in Figure 9, under each treatment, the contents of total N, soluble sugar, and cellulose, were positively correlated with the transcript levels of *PtrNINV5*, *PtrCWINV3*/*5*, *PtrVINV2*, *PtrHXK4*, *PtrPGM2*, and *PtrUGP1*. However, the total N content was negatively correlated with hemicellulose content, reducing sugar content, lignin content, and the transcript levels of *PtrNINV9*/*10*, *PtrCWINV2*, *PtrVINV1*/*3*, and *PtrSUS1*/*2*. The changes in reducing sugar content, starch content, lignin content, and hemicellulose content were positively correlated with the transcript levels of *PtrSUS2*, *PtrNINV10*, and *PtrVINV1*. In addition, the contents of reducing sugar, starch, lignin, and hemicellulose were significantly negatively correlated with cellulose content, but there was a positive correlation between total N content and cellulose content. The transcript levels of *PtrSUS3*/*5*, *PtrNINV1*/*2*/*3*/*5*/*12*, *PtrCWINV3*, *PtrVINV2*, *PtrHXK5*/*6*, *PtrPGM1*/*2*, and *PtrUGP1* were positively correlated with cellulose content, but negatively correlated with the contents of reducing sugar, starch, lignin, and hemicellulose.

### 2.9. Effects of PtrNINV12-knockout on Growth and Cell Wall Development of Poplar Seedlings

**Figure 10:**
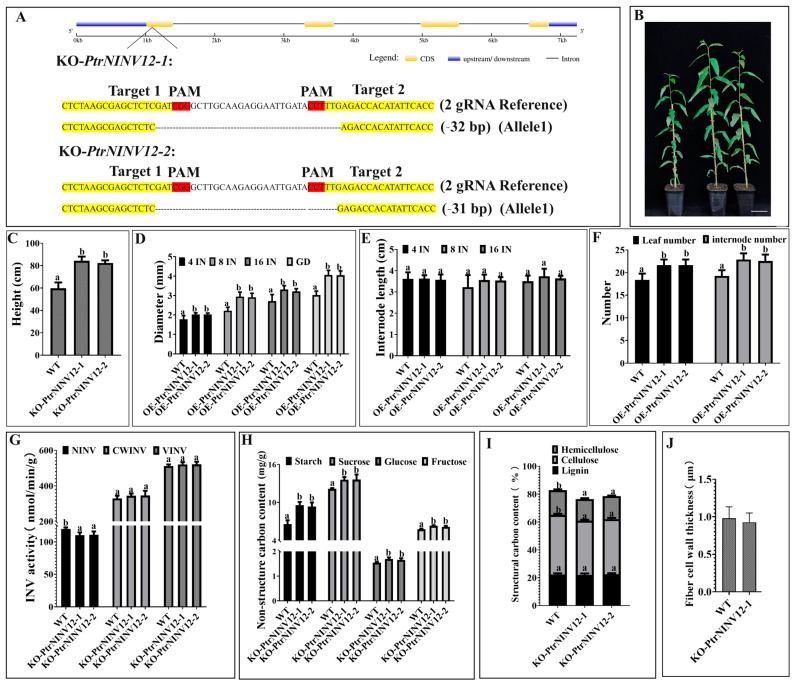
Morphology, structural C contents, and non-structural C contents of 3-month-old WT and *PtrNINV12-*knockout (KO-*PtrNINV12*) lines. (**A**) Two gRNAs were designed, and two homozygous lines (KO-*PtrNINV12*-1 and KO-*PtrNINV12*-2) were obtained. Coding sequences and upstream/downstream sequences are represented by yellow and blue lines, respectively. “-” represents base deletion, with the target and PAM sequence highlighted in yellow and red, respectively. (**B**) Morphology of KO-*PtrNINV12* and WT after cultivation for 3 months in a glasshouse. Scale bar = 10 cm. (**C**) Plant height. (**D**) Diameters of fourth (4 IN), eighth (8 IN), sixteenth (16 IN), and last internode (GD). (**E**) Internode length. (**F**) Number of leaves and internodes. (**G**) Invertase activity. (**H**) Non-structural C content. (**I**) Structural C content. (**J**) Fiber cell wall thickness at 8 IN. Same lowercase letters indicate insignificant differences between the lines tested using Duncan’s multiple range test with a significance level of *p* < 0.05.

**Figure 11:**
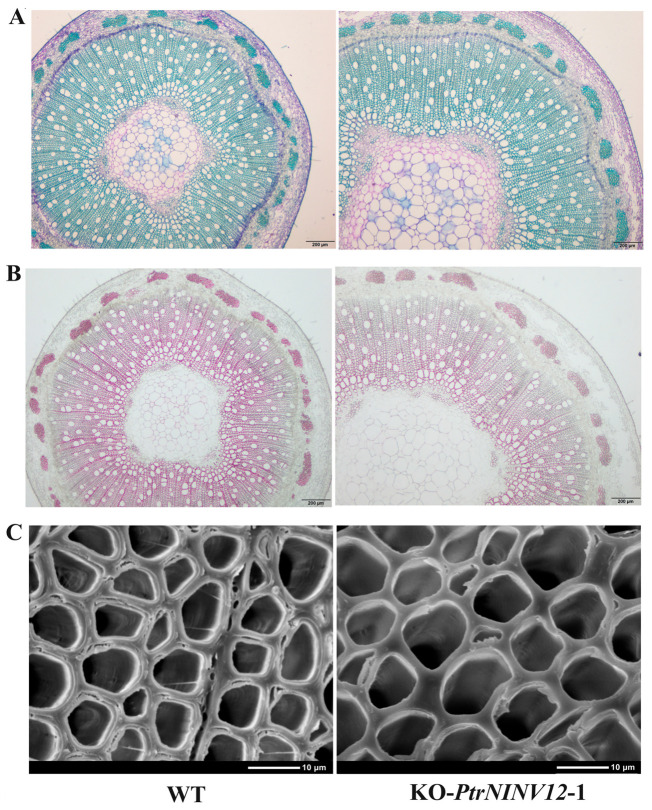
Stem secondary structure of 3-month-old WT and KO-*PtrNINV12* lines. (**A**) Toluidine blue staining of eighth internode (8 IN). Scale bar = 200 μm. (**B**) Phloroglucinol-HCl staining of 8 IN. Scale bar = 200 μm. (**C**) Scanning electron microscopy images of 8 IN. Scale bar = 10 μm.

To further demonstrate the relationship between genes and structural C, *PtrNINV12* was selected for transgenic analyses. The CRISPR/Cas9 vector of *PtrNINV12* was transformed into *P. trichocarpa*, and two knockout (KO) lines were obtained. As shown in Figure 10A, target 1 and target 2 were on the first exon. The two lines KO-*PtrNINV12*-1 and KO-*PtrNINV12*-2 were identified as homozygous mutants, with 32-bp and 31-bp deletions in the target gene, respectively. The plant height, number of leaves, and number of internodes were significantly higher in the KO lines than in WT, but internode length was not significantly changed (Figure 10B–F). As shown in Figure 10D, the stem diameter was also significantly greater in KO-*PtrNINV12* than in WT. To explore the role of *PtrNINV12* in cell wall synthesis, the activities of INVs (NINV, CWINV, and VINV) and the contents of non-structural carbohydrates and structural carbohydrates in stems of 3-month-old seedlings were measured (Figure 10G–I). Compared with WT, KO-*PtrNINV12* showed significantly decreased NINV activity and slightly increased CWINV and VINV activities (Figure 10G), as well as significantly increased contents of starch, sucrose, Glc, Fru in non-structural carbohydrates (Figure 10H). In addition, the cellulose and hemicellulose contents in structural carbohydrates were significantly reduced in KO-*PtrNINV12*, but there was no change in lignin content (Figure 10I). To observe the cell wall composition more intuitively, paraffin sectioning technology combined with toluidine blue and phloroglucinol-HCl staining (staining lignin as red) and scanning electron microscopy (SEM) analyses were conducted. Based on analyses of the structural carbohydrates content in WT and KO-*PtrNINV12* lines, the KO-*PtrNINV12*-1 line was selected for observation. The results showed that xylem was wider and lower lignin was deposited in cell walls in KO-*PtrNINV12* compared with WT (Figure 11A,B). The SEM analysis showed that the fiber cell walls were significantly thinner in KO-*PtrNINV12* than in WT (Figure 10J and Figure 11C). These results suggest that *PtrNINV12* plays a key role in non-structural C metabolism, mainly promoting the accumulation of cellulose and hemicellulose. The data of these indexes are listed in Table S7.

## 3. Discussion

N is an essential macronutrient, but it can also limit plant growth and development in natural soils [77]. Previous studies have shown that the cell walls of poplar are thickened in the elongation region under N treatments [66]. Other studies have shown that the cell wall thickness of *Populus × euramericana* (Dode) Guinier clone I-214 and *Populus alba* L. were reduced under elevated CO_2_ conditions, and that high CO_2_ and N fertilizer levels affected the distribution of C between mobile and structural carbohydrate fractions [68,69]. In fact, N metabolism and C metabolism are closely related [63,64,65]. The key enzymes involved in cell wall C metabolism are SUS, INV, HXK, PGM, and UGP; but it is unclear whether their gene expression and/or enzyme activities are affected by exogenous N and C supply. With the development of sequencing and bioinformatics technologies, the families of genes encoding these proteins have been identified and their structural characteristics have been analyzed in some plants, such as *Arabidopsis* and *O. sativa* [8,30,33,43,53,55]. In poplar, members of the *SUS* and *INV* gene families have been identified and analyzed [5,23], but members of the *HXK*, *PGM*, and *UGP* gene families have not. In addition, the structural characteristics and evolution of these gene families in poplar have not been analyzed, nor have their responses to exogenous N and C supply, nor the relationship between their transcript levels and the structural C content, been determined in previous studies. In this study, the contents of total N, soluble sugar, reducing sugar, starch, cellulose, lignin, and hemicellulose were determined after supplying exogenous N and C to poplar seedlings. Genome-wide analyses of *PtrSUS*, *PtrINV*, *PtrHXK*, *PtrPGM*, and *PtrUGP* genes were performed. The effects of N and C supply on the transcript levels of members of these gene families were determined by qRT-PCR. To further verify the relationship between the above genes and structural C contents, *PtrNINV12* was silenced. The results of these analyses provide a foundation for revealing the molecular mechanism by which SUS, INV, HXK, PGM, and UGP affect cell wall cellulose synthesis under the regulation of N and C supply.

### 3.1. Evolution and Structure

Amino acids determine protein structure, which in turn determines function. The amino acid sequences of PtrSUSs contain a conserved serine residue at the N-terminal, which is considered to be the phosphorylation site of SUS proteins [7,78]. In addition, PtrSUSs have conserved sucrose synthetase and glycosyltransferase domains, which are considered to be typical features of SUSs [9]. Our results and those of other studies confirmed that PtrNINVs have 12 conserved domains, and PtrCWINVs and PtrVINVs have 13 conserved domains [22]. The PtrHXKs contain four conserved domains: the phosphate 1, phosphate 2, sugar-binding, and adenosine domains. The phosphate 1, phosphate 2, and adenosine domains participate in ATP binding, and the sugar-binding regions are hexose-binding sites. These domains are specific to plant HXKs and are essential for their enzymatic function [79]. In addition, PtrHXKs include four additional peptide sequences (loops 1–4) that are induced to move upon binding of the sugar ligand. The sugar-binding sites and the nucleotide-binding site are completed or “pre-formed” by these loops [74]. The amino acid sequences of the reaction center and metal-binding sites of PtrPGM1 and 2 are T–A–S–H–N and D–G–D–A–D, respectively; and those of the reaction center and metal-binding sites of PtrPGM3 and 4 are S–A–S–H–N and D–G–D–G–D, respectively. As determined by comparison with *Arabidopsis* pPGMs, PtrPGM1 and 2 are cPGMs, and PtrPGM3 and 4 are pPGMs [80]. PtrUGPs contain nucleotide-binding, glycosylation, and proteolytic cleavage sites. Lys-360 is the catalytic center, and Lys-257 and Lys-322 are the catalytic binding sites [55]. 

Typically, gene families expand through tandem repetition and fragment repetition [81]. The number of genes in the *SUS*, *INV*, and *HXK* families vary greatly among plant species. For example, there are 14 *SUS*s in *B. juncea* but only 6 *SUS*s in *Arabidopsis* [8,9]. There are 32 *INV*s in *G. max* but only 17 *INV*s in *Arabidopsis* [22,82]. There are 14 *HXK*s in *P. bretschneideri* but only 6 *HXK*s in *Arabidopsis* [30,36]. Differences in the number of family members may be related to differences in the types of gene expansion [83]. In this study, 6 *PtrSUS*s, 20 *PtrINV*s, and 6 *PtrHXK*s were identified in *P. trichocarpa* (Table S2). Fragment replication relationships were detected for *PtrSUS1/2* and *PtrSUS6/7*, and for seven *PtrINV*s, whereas tandem replication relationships were detected for *PtrCWINV4/5* and *PtrCWINV1/2*. Similarly, fragment replication relationships were detected for *PtrHXK2/5* and *PtrHXK1/6* (Figure 3 and Figure S3). In most plant species, the *PGM* and *UGP* gene families tend to be small, containing one to four genes [42,43,44,45]. In this study, four *PGM*s and two *UGP*s were identified in the *P. trichocarpa* genome (Table S2). In addition, we detected fragment replication relationships for *PtrPGM1/2* and *PtrPGM3/4*, and for the two *PtrUGP*s. Together, our results show that the *INV* gene family has expanded by fragment replication and tandem replication events, while the *SUS*, *HXK*, *PGM*, and *UGP* gene families have expanded by fragment replication. In addition, *SUS*, *INV*, *HXK*, *PGM*, and *UGP* genes had 12, 33, 20, 8, and 5 homologous gene pairs, respectively, between *P. trichocarpa* and *G. max*; and 4, 10, 3, 6, and 0 homologous gene pairs, respectively, between *P. trichocarpa* and *Arabidopsis* (Figure 4). These results show that *SUS*, *INV*, *HXK*, *PGM*, and *UGP* genes have evolved differently between monocots and dicots.

A previous study showed that SUSs are highly conserved in many dicotyledonous and monocotyledonous plants [84]. However, we detected some differences in exon–intron structure among the groups. For example, in the SI group (PtrSUS1, 2 and 3), the 5^th^ and 11^th^ exons of *PtrSUS1* and *PtrSUS2* were split by introns into exons 5/6 and exons 12/13, respectively, in *PtrSUS3*. In addition, *PtrSUS1* and *PtrSUS2* had longer UTRs at the 5′ end, and *PtrSUS7* had two more exons at the 3′ end. Further research is required to explore the significance of intron splitting, extra exons at the 3′ end, and longer UTRs. Eight acid invertase genes were identified in this study. Our results showed that *PtrVINV1* has no introns, but it retains 13 conserved motifs of the acid invertase subfamily, suggesting that *PtrVINV1* may have lost introns during evolution. *PtrCWINV1*/*2* have six exons and one fewer mini exon (encoding three amino acids) than the other acid invertase genes, suggesting that *PtrCWINV1*/*2* may have lost some functions during evolution. The mini exon is a typical structural feature of acid invertase genes in plants and is the smallest exon found in plants so far [83,85]. A total of 12 *PtrNINV*s were identified, with PtrNINV1–6 in the β subgroup encoded by genes containing six exons, and PtrNINV7–12 in the α subgroup encoded by genes containing four exons (Figure 2 and Figure S2). Our results are basically consistent with those of other studies [86,87]. Our analyses revealed that the exon–intron structure of the six *PtrHXK* genes is similar, all of them containing nine exons, but we detected differences in exons 1, 5, 6, 7, and 9 (Figure 2 and Figure S2). The exon–intron structure of the two *PtrUGP*s was found to be highly conserved, both containing 21 exons, with only 2–3 bp variations in exons 2, 3, and 8 (Figure S2); but *PtrUGP1* lacks a UTR sequence. The exon–intron structure of the four *PtrPGM*s was found to be similar, with 18 exons in *PtrPGM1*/*2* (SI) and 22 exons in *PtrPGM3*/*4* (SII) (Figure 2 and Figure S2). Compared with *PtrPGM1*/*2*, *PtrPGM3*/*4* have gained or lost introns [84]. PtrPGM1/2 were identified as cytoplasmic proteins, and PtrPGM3/4 as plastidic proteins. Further research is required to determine whether the longer N-terminus of PtrPGM3/4 is a signal peptide related to localization.

### 3.2. Expression Patterns of PtrSUS, PtrINV, PtrHXK, PtrPGM, and PtrUGP Genes

Comprehensive expression analysis of all gene family members is helpful to understand their function. Previous studies have not comprehensively analyzed the transcript profiles of *PtrSUS*, *PtrINV*, *PtrHXK*, *PtrPGM*, and *PtrUGP* genes in different tissues [23,88]. In this study, we detected the highest transcript levels of *PtrSUS1/2/5/6*/*7* in the stems, and the highest transcript level of *PtrSUS3* in the mature leaves (Figure 7). In *P. tremula* × *P. tremuloides*, inhibition of *SUS1* and *2* expressions led to a decrease in wood density and cellulose content because of cell wall loosening [16]. Inhibition of *SUS* expression in *G. arboreum* resulted in significant decreases in cellulose and callose contents in seed endosperm cells, along with delayed fiber initiation [15]. These studies suggest that *PtrSUS1/2/5/6*/*7* may play roles in cellulose synthesis in the poplar stem cell wall. Analyses of *A. thaliana* revealed high transcript levels of *AtSUS1* and *4* in mature leaves, and *atsus1* and *atsus4* mutants showed slow growth and increased Glc and Fru content in leaves, especially in the absence of oxygen [17]. *PtrSUS3* may have similar functions to those of *AtSUS1* and *4*.

In *Arabidopsis*, *cinv1cinv2* lost anisotropic growth characteristics, with significantly reduced cellulose content, suggesting that CINVs play a central role in cellulose biosynthesis and C allocation [27]. In *P. tremula* × *tremuloides*, a 38–55% decrease in activity of CINV12, which was highly expressed in stems, resulted in a 9–13% decrease in the crystal cellulose content, providing further evidence that CINVs play an important role in cellulose biosynthesis [29]. Therefore, it is reasonable to speculate that the high transcript levels of *PtrNINV1/7/8/9*/*12* in poplar stems may be related to sucrose decomposition during cellulose synthesis. Heterologous expression of *PhCWINV1, PhCWINV4*, and *PhCWINV7* (which are highly expressed in stems of *Phyllostachys bambusoides* Sieb. et Zucc. f. lacrima-deae Keng f. et Wen) in *Arabidopsis* led to dwarfism [89], suggesting that acid invertases play important roles in internode elongation. Therefore, we speculated that *PtrCWINV2* may be related to stem internode elongation in poplar. Inhibition of *GhVINV1* expression in *G. arboreum* repressed fiber initiation in seeds [26], leading to a significant decrease in cellulose content, suggesting that VINVs may be involved in cellulose synthesis [25]. We observed that *PtrVINV2* was highly expressed in poplar stems, and this may be related to sucrose decomposition in the vacuole during cellulose synthesis.

PtrHXK1/6 and AtHXK1/2 belong to the SII group and showed similar expression patterns (Figure 2 and Figure 7). Studies on *Arabidopsis* revealed that *AtHXK1* transcripts were abundant in all sampled tissues, whereas *AtHXK2* transcripts were only detected in the leaves [30]. In *Arabidopsis*, *gin2-1* (the *AtHXK1* mutant) showed reduced stem and root length, decreased leaf expansion and auxin sensitivity, and increased apical dominance, sensitivity to cytokinin, and delayed flowering and senescence [90]. We speculate that the functions of *PtrHXK1* or *6* may be similar to those of *AtHXK1* and *2*.

In this study, we found that the highest transcript levels of *PtrPGM1*/*2*, encoding cPGMs, were in the stems, whereas the highest transcript levels of *PtrPGM3*/*4*, encoding pPGMs, were in the leaves (Figure 7). In *Arabidopsis*, *pgm2/3* (mutants of *PGM2/3* encoding cPGMs) showed altered cell wall composition and reduced root length compared with WT [48]. The knock-down of *pPGM* in *S. tuberosum* using antisense technology resulted in significantly decreased starch content in tubers, but significantly increased sucrose content [52]. The complete loss of *cPGM* and *pPGM* in *Arabidopsis* resulted in dwarfed plants, premature death, wilted flower buds, and serious negative effects on plant growth and development [48]. Therefore, we speculate that cytosolic *PtrPGM1/2* may be involved in sugar distribution for cell wall synthesis, and plastidic *PtrPGM3/4* that are mainly expressed in leaves may be related to starch metabolism.

In *Arabidopsis*, *AtUGP1* and *2* were found to be highly expressed in stems. The *atugp1* and *atugp2* mutants showed no growth defects, whereas the *atugp1/atugp2* double mutant showed severe growth defects and down-regulated expression of genes encoding enzymes involved in cellulose and callose synthesis [53]. In this study, the highest transcript levels of *PtrUGP1*/*2* were in stems (Figure 7), suggesting that *PtrUGP1*/*2* might be involved in the synthesis of cellulose and other carbohydrates. In conclusion, the genes that were highly expressed in stems of *P. trichocarpa*, namely *PtrSUS1/2/5/6/7*, *PtrNINV1/7/8/9/12*, *PtrVINV2*, *PtrCWINV2*, *PtrHXK1/2/3/5*, *PtrPGM1/2*, and *PtrUGP1/2*, may be involved in the supply of sugars needed for cellulose synthesis in stem cell walls. Further studies are required to elucidate the specific regulatory functions of these genes in the supply of sugars for cellulose synthesis. 

### 3.3. Changes in Structural C Content and Gene Expression Patterns under N and C Treatments

In this study, we determined the effects of supplying N and C at various concentrations on various indexes and gene expression in poplar seedlings. As the N concentration increased, the cellulose content in stems increased significantly, while the hemicellulose and lignin contents decreased (Figure 1 and Figure 9). Under N1 conditions, high C treatment would not cause the change in structural C content, but high C treatment based on different N treatments would strengthen the increase in degree of cellulose content with the increase in N concentration. This result might indicate that high CO_2_, while having little effect on structural C, will increase N uptake by plants [69]. In this study, we observed that the members of each gene family showed complex expression patterns in response to exogenous N and C (Figure 8 and Figure 9). For example, the transcript levels of *PtrSUS1/6*, *PtrNINV3/7/8/9/11*, *PtrCWINV3/4/5*, *PtrVINV2*, and *PtrHXK1/6* were significantly lower in the N0.1-C8 and N0.1-C4 treatments than in the control, and lower in the N0.1-C4 treatment than in the N0.1-C8 treatment (Table S6). The transcript levels of *PtrSUS2*/*5* and *PtrVINV3* were significantly higher in the N0.1-C8 treatment than in the control, but significantly lower in the N0.1-C4 treatment than in the control. The transcript level of *PtrCWINV2* was also significantly higher in the N0.1-C8 treatment than in the N0.1-C4 treatment. The transcript levels of *PtrHXK2* and *PtrUGP2* showed no obvious changes in the N0.1-C4 treatment than in the control, but were significantly increased in the N0.1-C8 treatment than in the control. These results indicated that exogenous C at a high concentration not only reduced the degree of inhibition by low-N on gene transcript levels, but also increased the transcript levels of genes that were not affected by low N. At the same time, it also made the already increased gene expression level more significant. Our results support the hypothesis that exogenous C increases N uptake by plants [69]. Further research is needed to investigate the involvement of individual genes within these five families in changes in the contents of structural C under various N and C conditions.

### 3.4. Effects of PtrNINV12-Knockout on Contents of Structural C and Non-Structural C

The expression pattern of *PtrSUS3/5*, *PtrNINV1/2/3/5/12*, *PtrCWINV3*, *PtrVINV2*, *PtrHXK5/6*, *PtrPGM1/2*, and *PtrUGP1* were positively correlated with changes in cellulose content (Figure 9), suggesting that these genes may play essential roles in cellulose synthesis. *PtrNINV12* showed the highest transcript levels in stems, so it was selected for further analysis. Compared with WT, the *PtrNINV12*-deficient line had significantly lower cellulose and hemicellulose contents, and thinner fiber cell walls (Figure 10I,J). The increased Glc and Fru levels in KO-*PtrNINV12* were perplexing (Figure 10H), because reduced NINV activity was expected to result in decreased levels of its products. We speculated that, under sucrose surplus, the activities of INVs in other compartments and organelles such as the cell wall, vacuole, plastids, and mitochondria might contribute to Glc and Fru generation in non-cytosolic compartments. We speculated that soluble sugar accumulated in KO-*PtrNINV12* because they were not used for the production of UDP-Glc. In addition, cell wall integrity sensing, phosphatidylinositol signaling, and hormone regulation may also influence C metabolism in unknown ways [91]. In KO-*PtrNINV12*, the starch content was significantly increased (Figure 10H), and this was closely related to decreased cellulose content (Figure 10I). In *Arabidopsis*, it has been shown that C resources are distributed between cell wall and starch synthesis, so that defective cellulose biosynthesis leads to the diversion of C resources into starch biosynthesis [92]. In addition, increased soluble sugar levels may lead to increased starch content by inhibiting starch catabolism. In summary, the changes in structural and non-structural C metabolites in KO-*PtrNINV12* mutants increase the credibility of the above speculation.

### 3.5. PtrSUS, PtrINV, PtrHXK, PtrPGM, and PtrUGP Family Genes Participate in the Process of Exogenous N and C Affecting Structural C Synthesis

**Figure 12:**
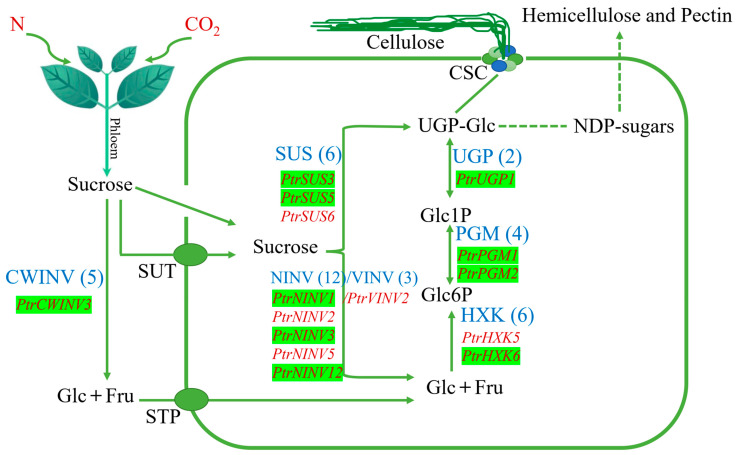
Model showing how N and C concentrations affect sucrose metabolism in the cell wall. Model graph relation refers to existing studies [1]. Genes that contribute to changes in carbohydrate metabolism under N and C treatments are shown in red font, and numbers of genes in *PtrSUS*, *PtrINV*, *PtrHXK*, *PtrPGM*, and *PtrUGP* families are shown in blue font. Potential co-evolved genes are shown with green highlighted background.

In plants, SUS, INV, HXK, PGM, and UGP are the key enzymes for sucrose metabolism, and structural C are the final storage forms of sucrose [1]. Secondary growth occurs in poplar, making it an ideal material for studying the synthesis of structural C. In this study, exogenous C and N affected the contents of structural C in *P. trichocarpa* (Figure 1G–I). The results of correlation analyses showed that the transcript levels of *PtrSUS3/5*, *PtrNINV1/2/3/5/12*, *PtrCWINV3*, *PtrVINV2*, *PtrHXK5/6*, *PtrPGM1/2*, and *PtrUGP1* were correlated with the structural C contents in different treatments. Therefore, based on the results of the correlation analyses and tissue-specific transcript profiles of various genes (Figure 7 and Figure 9), *PtrSUS3*, *PtrNINV12*, *PtrCWINV3*, *PtrVINV2*, *PtrHXK5*, *PtrPGM1*, and *PtrUGP1* were selected to verify the speculation. Although a knockout line was successfully obtained only for *PtrNINV12*, knockout of *PtrNINV12* resulted in change in the contents of structural non-structural C. These results demonstrate that *PtrSUS3/5*, *PtrNINV1/2/3/5/12*, *PtrCWINV3*, *PtrVINV2*, *PtrHXK5/6*, *PtrPGM1/2*, and *PtrUGP1* are involved in the synthesis of structural C. In addition, in the natural environment, the evolution of protein is an important way of biological evolution, and has usually cooperativity because of functional and natural selection. To adapt to changes in the external environment, related genes involved in the same biological pathway may co-evolve [93]. In our study, *PtrSUS3/5/6/7*, *PtrNINV1/3/4/12*, *PtrCWINV3*, *PtrHXK3/6*, *PtrPGM1–4*, and *PtrUGP1–2* showed similar transcript profiles and exhibited positive correlations with each other during adaptation to changes in external C and N conditions, so we speculated that they may have co-evolved. However, this hypothesis needs to verified by further research. In the future, we will perform functional validation of these genes and explore their transcriptional responses to C and N treatments. Elucidating the molecular modules affected by C and N during poplar cell wall formation will be helpful for poplar research and breeding. In conclusion, as shown in Figure 12, under the influence of exogenous N and C, the expression pattern alterations of gene family members of key enzymes in the sucrose metabolic pathway were observed. Additionally, there is the co-evolution potential among certain genes within this process.

## 4. Materials and Methods

### 4.1. Analysis of SUS, INV, HXK, PGM, and UGP Family Members in P. trichocarpa

The amino acid sequences of the AtSUSs, AtINVs, AtHXKs, AtPGMs, and AtUGPs were downloaded from the *Arabidopsis* Information Resource database (https://www.arabidopsis.org, accessed on 2 July 2023). The Pfam database (http://pfam.xfam.org, accessed on 2 July 2023) was used to search for the Hidden Markov Model (HMM) profiles of the five gene families based on an expected value (E-value) cutoff of 1 × 10^−5^ in HMMER 3.3.2 (http://hmmer.org, accessed on 3 July 2021) to find corresponding genes in the *P. trichocarpa* genome. Furthermore, the candidate genes were identified by conducting BlastP searches on NCBI (https://www.ncbi.nlm.nih.gov, accessed on 5 July 2023) using AtSUS, AtINV, AtHXK, AtPGM, and AtUGP as query sequences. Finally, certain candidate genes identified through both methods were manually excluded based on reported conserved domains in plants. In previous studies, it was reported that there were 7 *PtrSUSs* in *P. trichocarpa*, considering alternative splicing genes as one gene [5]. However, with the database update, we believe that the deletion of *PtrSUS4* was reasonable. While earlier research identified 24 *PtrINVs* [23], *PtrNINV13*–*16* were found lacking a complete *PtrINVs* structure and have since been removed from the latest database. Ultimately, 6 *SUSs*, 20 *INVs*, 6 *HXKs*, 4 *PGMs*, and 2 *UGPs* family members were identified in *P. trichocarpa*. They are named as *PtrHXK1*–*6*, *PtrPGM1*–*4*, and *PtrUGP1*–*2* based on their chromosomal locations. The nomenclature of *PtrSUSs* and *PtrINVs* family members adheres to established conventions. The basic characteristics of PtrSUSs, PtrINVs, PtrHXKs, PtrPGMs, and PtrUGPs (molecular weight, isoelectric point, amino acid number, aliphatic index, GRAVY, and sub-cellular localization) were analyzed using the ExPASy website (http://www.expasy.org/, accessed on 1 June 2023) [94].

### 4.2. Analysis of Gene Structure, Conserved Motifs, Multiple Sequence Alignment, Phylogenetic Trees

The *P. trichocarpa* v4.1 (https://phytozome-next.jgi.doe.gov/info/Ptrichocarpa_v4_1, accessed on 5 June 2023) database was used to download CDS, protein, and genome sequence of members in five gene families. Simultaneously, the Gene Structure Display Server (GSDS2.0, http://gsds.cbi.pku.edu.cn, accessed on 6 June 2023) [95] and Multiple Em for Motif Elicitation v4.11.3 (http://meme-suite.org/tools/meme, accessed on 6 June 2023) [96] were used to analyze their intron-exon composition and predict conservative motifs (set the number of motifs to 10 and keep the rest as default). The BioEdit v7.2.5 software was used to perform multiple alignments of amino acid sequences and check the conserved motifs. The MEGA v7.0 software was used to construct the phylogenetic tree by Neighbor-Joining (NJ) method (Number of bootstrap replications = 1000) [97].

### 4.3. Protein Secondary, Tertiary Structure Prediction, and Chromosome Distribution

The location information of genes was retrieved from the *P. trichocarpa* v4.1 database. Their chromosomal location and gene replication events were mapped and analyzed with the MG2C tool (MapGene2Chrom web v2, http://mg2c.iask.in/mg2c_v2.0, accessed on 10 June 2023) and MCScanX toolkit (default parameters of python version) [98]. The secondary and tertiary structures of proteins were predicted by the online tool NPSA (https://npsa-prabi.ibcp.fr, accessed on 10 June 2023) and SWISS-MODEL (https://swissmodel.expasy.org, accessed on 10 June 2023), respectively.

### 4.4. Plant Materials, Growing Conditions, and Treatments

The *P. trichocarpa* of genotype Nisqually-1 (an ecologically tree species of widespread ranging from Alaska to northern California [99]) used in this study was obtained from the greenhouse of the Northeast Forestry University, Harbin, China. Seedlings from the same clone were rooted in water, subsequently transplanted into pots filled with fine soil, and cultivated for a duration of 3 months in a greenhouse subjected to a 16-h light/8-h dark photoperiod at 22 ± 2 °C. Samples were divided into roots, upper stem (1–4), lower stem (5–14), mature leaves (leaves corresponding to the upper stem), and young leaves (leaves corresponding to lower stem), which were immediately collected in liquid nitrogen and stored at −80 °C for subsequent experiments. 

Seedlings of the same clone were rooted in water, and then (approximately 10 cm height) transplanted into hydroponic box and continued to be cultured for 14 days (approx 17 cm height) in a greenhouse under a 16-h light/8-h dark photoperiod at 22 ± 2 °C. During this period, the seedlings were supplied with a refreshed nutrient solution twice a week using 1/2 N-free Hoagland (containing 1 mM NH_4_NO_3_) [100]. Plant materials were cultured with 1/2 N-free Hoagland nutrient solution in an artificial climate chamber, then supplied with following 6 C and N interaction treatments by changing the concentration of CO_2_ and NH_4_NO_3_: 0.1 mM NH_4_NO_3_ and 400 ppm CO_2_ (N0.1-C4), 1 mM NH_4_NO_3_ and 400 ppm CO_2_ (N1-C4), 5 mM NH_4_NO_3_ and 400 ppm CO_2_ (N5-C4), 0.1 mM NH_4_NO_3_ and 800 ppm CO_2_ (N0.1-C8), 1 mM NH_4_NO_3_ and 800 ppm CO_2_ (N1-C8), 5 mM NH_4_NO_3_ and 800 ppm CO_2_ (N5-C8) [66,101,102]. The treatments lasted for 28 days. The 1 mM NH_4_NO_3_ (N1) and 400 ppm CO_2_ (C4) treatments were used as control (to simulate normal plant growth conditions). Then, the samples were immediately harvested in liquid nitrogen and stored at −80 °C for subsequent experiments.

### 4.5. RNA Extraction and qRT-PCR Analysis

Total RNA was extracted from different tissues using CTAB [103]. Subsequently, PrimeScript TM RT reagent Kit (containing gDNA Eraser) (Takara Bio, Dalian, China) was used to form cDNA. PtrUBQ7 was chosen as the internal reference gene [104] because of its stable expression levels. The qRT-PCR was conducted using UltraSYBR Mixture (Low ROX) (CWBIO, Beijing, China) on the LightCycler 480 II (Roche, Basel, Switzerland). The detailed procedure followed the manufacturer’s instructions. The relative expression level was calculated using 2^−ΔΔCT^ method with three biological replicates [105]. All gene primers were listed in Table S8. Tbtools v1.120 software [106] was used to generate heatmaps of gene expression.

### 4.6. Biochemical Analyses

Plant height, internode (IN) length (4 IN, 8 IN, 16 IN), and diameter (4 IN, 8 IN, 16 IN, ground diameter) were measured with a ruler and vernier caliper, respectively. Chlorophyll content of the fourth leaf was determined with a chlorophyll analyzer (TYS-4N, Beijing Jinkolida Electronic Technology Co., Ltd., Beijing, China). In each treatment, 9 plants were measured, and each plant was measured 3 times for chlorophyll content. The contents of soluble sugar, reducing sugar, starch, sucrose, Fru, Glc, hemicellulose, and INVs activity were all determined using reported methods kits (Suzhou Kming Biotechnology Co., Ltd., Suzhou, China). Lignin and cellulose were determined using reported methods as previously described [107,108]. The total N determination process is as follows: The whole sample was ground in a high-speed grinder (Wuxi Jiuping Instrument Co. Ltd., Wuxi, China), then digested in H_2_SO_4_-H_2_O_2_ for 2 h. Finally, the total N content was determined by the Kjeltee 2300 analyzer (Foss Tecator AB, Höganäs, Sweden). The stem tissue was utilized for the determination of lignin, cellulose, hemicellulose, and INVs activities, while other biochemical indexes were determined in roots, stems, and leaves. Three biological replicates were set for reliable results.

### 4.7. The Acquisition of Transgenic Plants

The CRISPR online website (http://crispr.hzau.edu.cn/CRISPR2/, accessed on 2 July 2023) was used to obtain efficient gRNA target sites. The Cas9/gRNA constructs were cloned by amplifying the PCR fragment using pCBC-DT1T2 as a template. Subsequently, the purified PCR fragment and pKSE401 plasmid were set up for the Golden Gate reaction using *BsaI* and T4 ligase. Agrobacterium-mediated transformation of *P. trichocarpa* was then conducted. To figure out the editing methods, the genomic DNA was used for PCR amplification with gene-specific primers spanning target sites. The PCR products were sent for NGS (https://doi.org/10.1007/s11427-018-9402-9, accessed on 2 September 2023) at State Key Laboratory of Rice Biology using the Hi-TOM platform (China National Rice Research Institute, Chinese Academy of Agricultural Sciences, Hangzhou, China). All primers were listed in Table S9.

### 4.8. Microscopy Analyses

For light microscopy, the 8th internode of *Populus* was fixed, embedded in paraffin, sectioned, and stained with toluidine blue and phloroglucinol-HCl. For scanning electron microscopy (SEM), free-hand cross-sections of fresh 8th internode samples were coated with gold (Au), then transferred to an SEM (S-4800; Hitachi, Tokyo, Japan) chamber, and the fiber cell wall thickness was analyzed.

### 4.9. Statistical Analysis

The data were subjected to one-way analysis of variance with Duncan’s multiple range test at *p* < 0.05 using SPSS 22.0 (IBM, Armonk, NY, USA). Correlation plots were generated by Origin software (Version: Pro 2022b SR1). 

## 5. Conclusions

In this study, 6 *PtrSUS*, 20 *PtrINV*, 6 *PtrHXK*, 4 *PtrPGM*, and 2 *PtrUGP* genes were identified in *P. trichocarpa*. Tandem and fragment replication events have contributed to the amplification of the *PtrINV* family, whereas the *PtrSUS*, *PtrHXK*, *PtrPGM*, and *PtrUGP* families have expanded by fragment replication. All conserved motifs were basically consistent within each protein family or subfamily. Members of each family were found to be differently expressed among tissues and responded to C and N at various concentrations. Changes in the transcript levels of *PtrSUS3/5*, *PtrNINV1/2/3/5/12*, *PtrCWINV3*, *PtrVINV2*, *PtrHXK5/6*, *PtrPGM1/2*, and *PtrUGP1* were consistent with changes in cellulose content, suggesting that they are key genes involved in the synthesis of structural C. The changes in the contents of structural and non-structural C metabolites in the KO-*PtrNINV12* mutant provide support for this speculation. The results of our study represent a genome-wide analysis of gene families related to sucrose metabolism, and lay the foundation for further research on the roles of *SUS*, *INV*, *HXK*, *PGM*, and *UGP* genes in cell wall cellulose synthesis under regulation by N and C.

## Data Availability

All experiments and data are available in this article and the Supplementary Materials.

## Supplementary Materials

The following supporting information can be downloaded at: https://www.mdpi.com/article/10.3390/ijms242417277/s1.
