# Supplementary material for: Structure and Expression Analysis of PtrSUS, PtrINV, PtrHXK, PtrPGM, and PtrUGP Gene Families in Populus trichocarpa Torr. and Gray
Source: Int J Mol Sci. 2023 Dec 8;24(24):17277. doi: 10.3390/ijms242417277 (PMC10743687; doi:10.3390/ijms242417277)
Supplement: Supplementary file 1 [file ijms-24-17277-s001.zip › Figure S1.pdf]

A

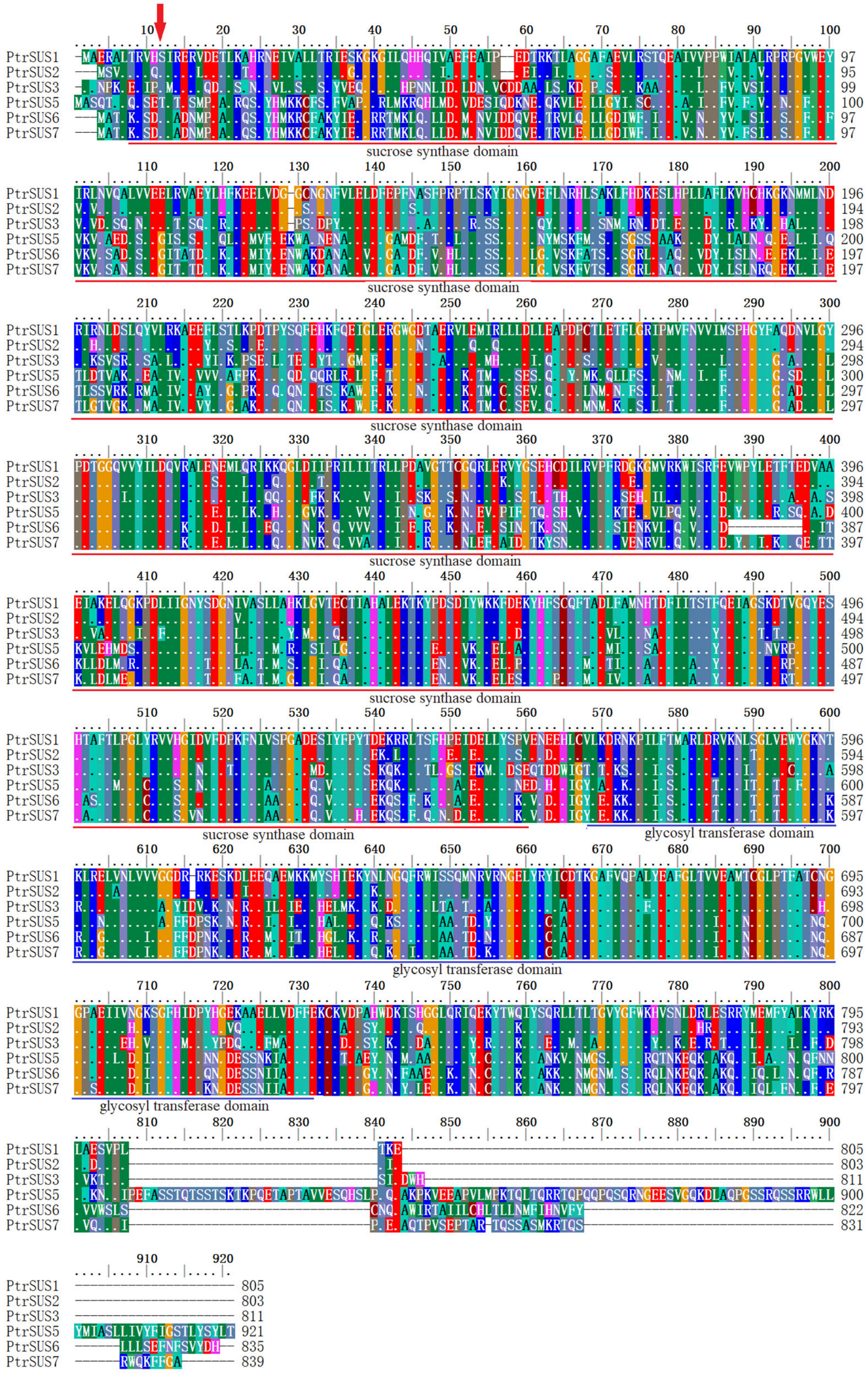

PtrSUS1
PtrSUS2
PtrSUS3
PtrSUS5
PtrSUS6
PtrSUS7
sucrose synthase domain
glycosyl transferase domain

B

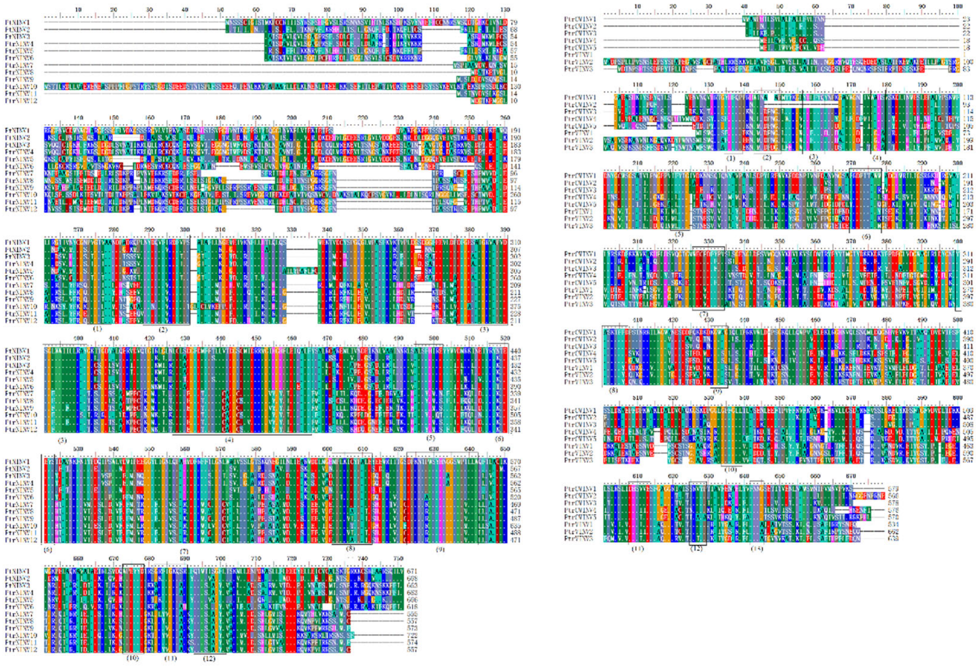

C

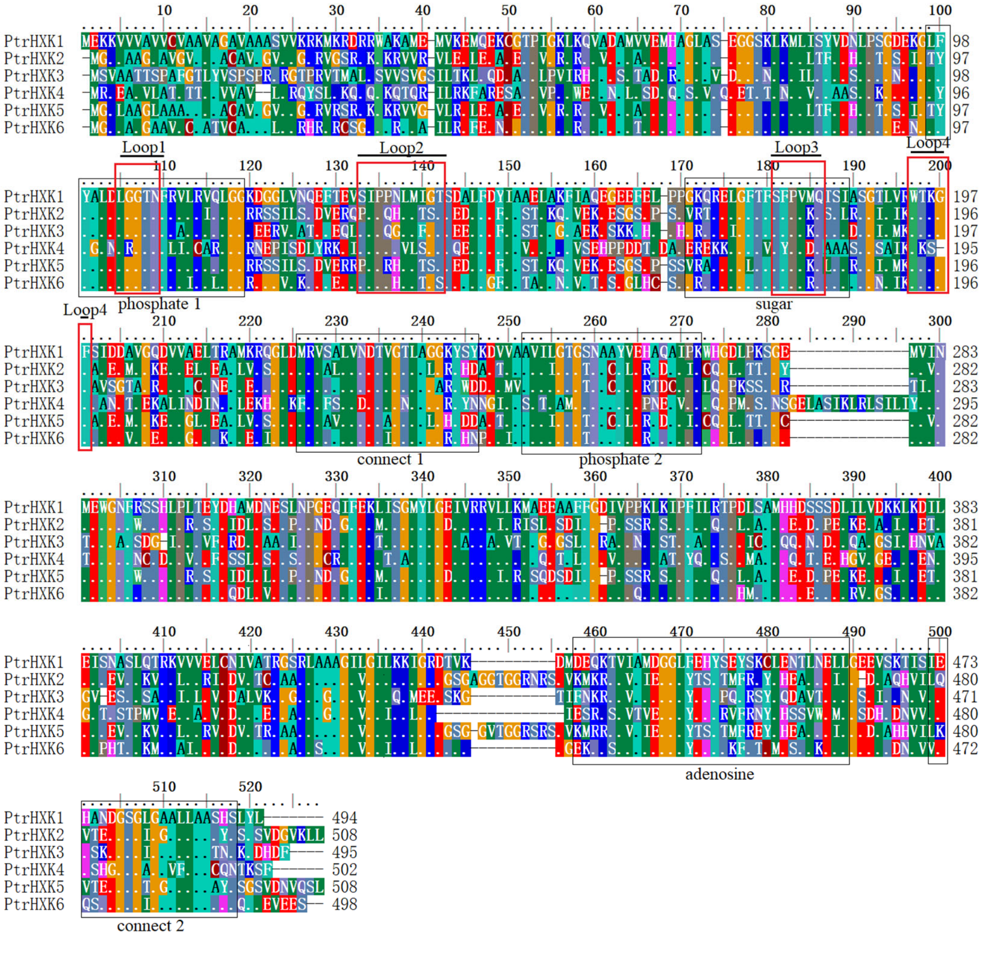
PtrHXK1
PtrHXK2
PtrHXK3
PtrHXK4
PtrHXK5
PtrHXK6
Loop1
Loop2
Loop3
Loop4
phosphate 1
sugar
connect 1
phosphate 2
adenosine
connect 2

D

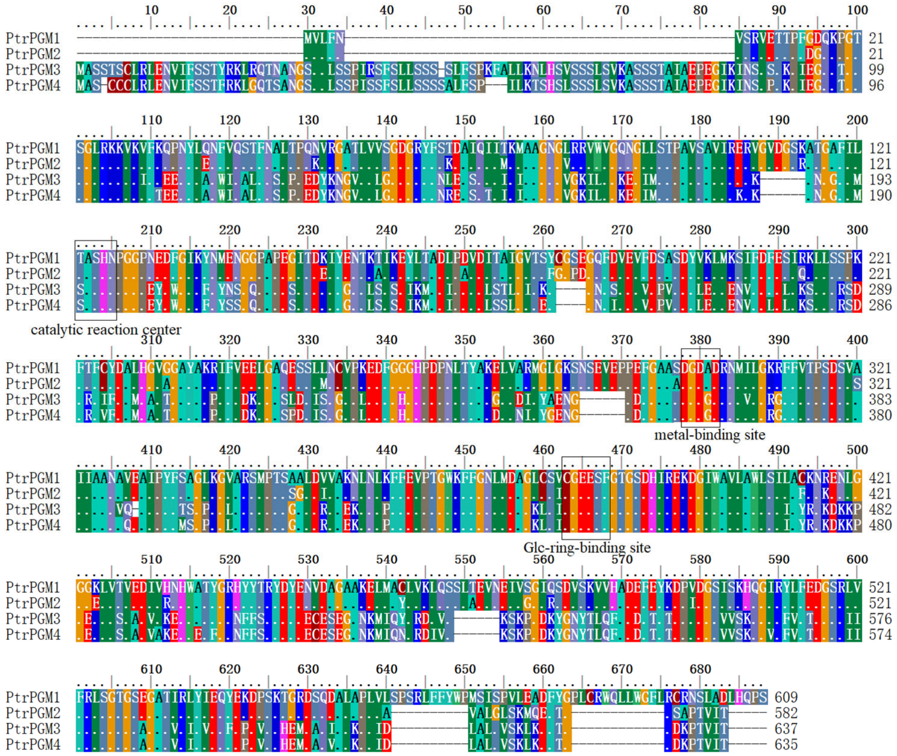

E

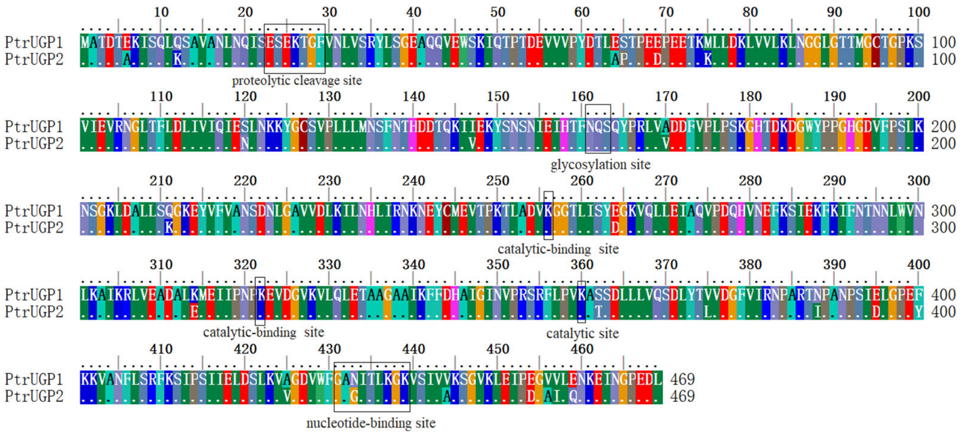

Figure S1. Multiple alignment of PtrSUS (A), PtrINV (B), PtrHXK (C), PtrPGM (D) and PtrUGP (E). Conserved motifs are represented by boxes and underline.
