# Supplementary material for: Structure and Expression Analysis of PtrSUS, PtrINV, PtrHXK, PtrPGM, and PtrUGP Gene Families in Populus trichocarpa Torr. and Gray
Source: Int J Mol Sci. 2023 Dec 8;24(24):17277. doi: 10.3390/ijms242417277 (PMC10743687; doi:10.3390/ijms242417277)
Supplement: Supplementary file 1 [file ijms-24-17277-s001.zip › Figure S2.pdf]

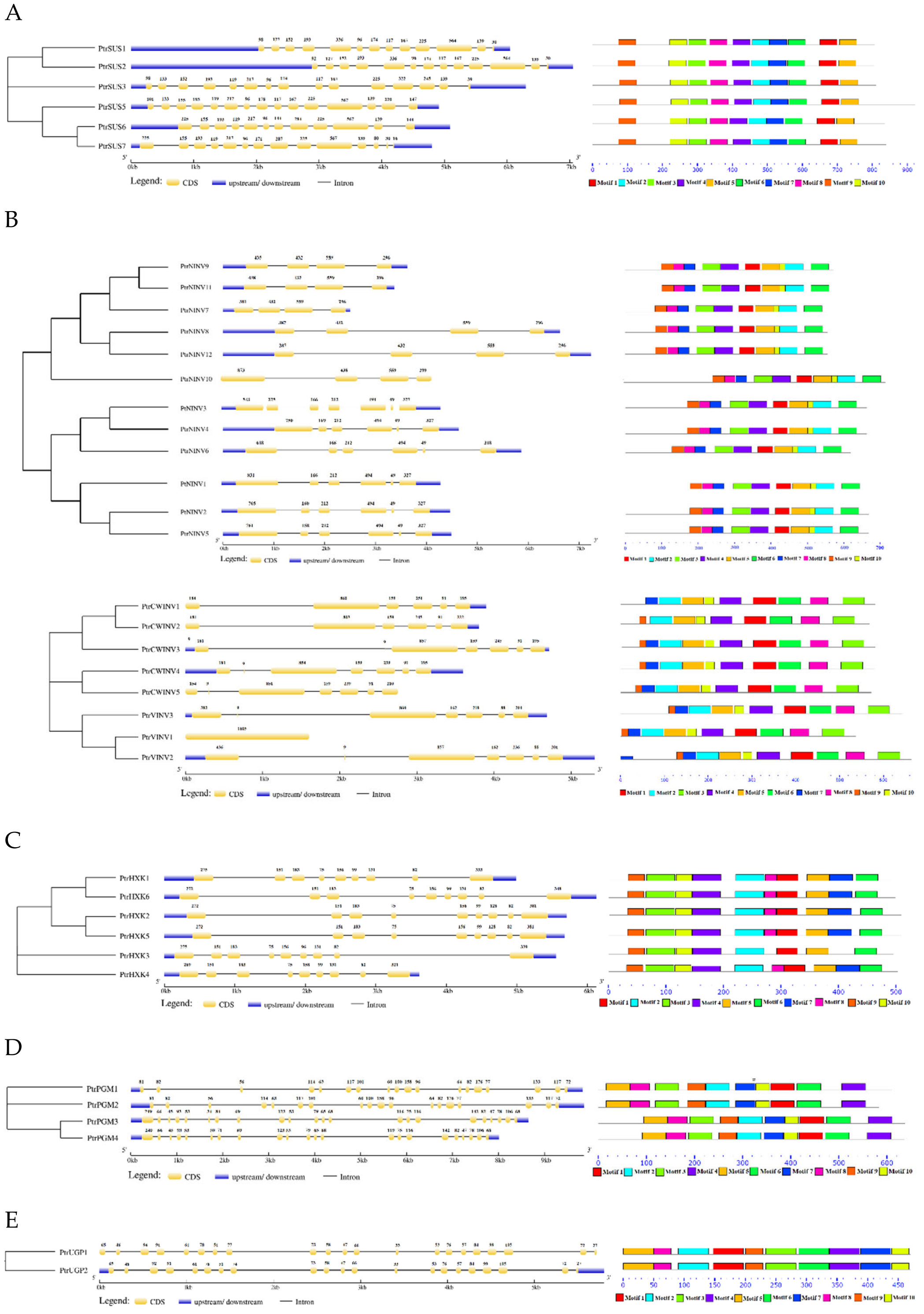

Figure S2. Phylogeny and structure analysis of *PtrSUS* (A), *PtrINV* (B), *PtrHXK* (C), *PtrPGM* (D) and *PtrUGP* (E) genes. Phylogenetic tree was generated according to sequences of proteins using the neighbor-joining method. Structure of corresponding genes, CDSs and the upstream/ downstream sequences are represented by yellow and blue lines, respectively. Motif sequences were predicted by online MEME tool;
