# Supplementary material for: Structure and Expression Analysis of PtrSUS, PtrINV, PtrHXK, PtrPGM, and PtrUGP Gene Families in Populus trichocarpa Torr. and Gray
Source: Int J Mol Sci. 2023 Dec 8;24(24):17277. doi: 10.3390/ijms242417277 (PMC10743687; doi:10.3390/ijms242417277)
Supplement: Supplementary file 1 [file ijms-24-17277-s001.zip › Figure S3.pdf]

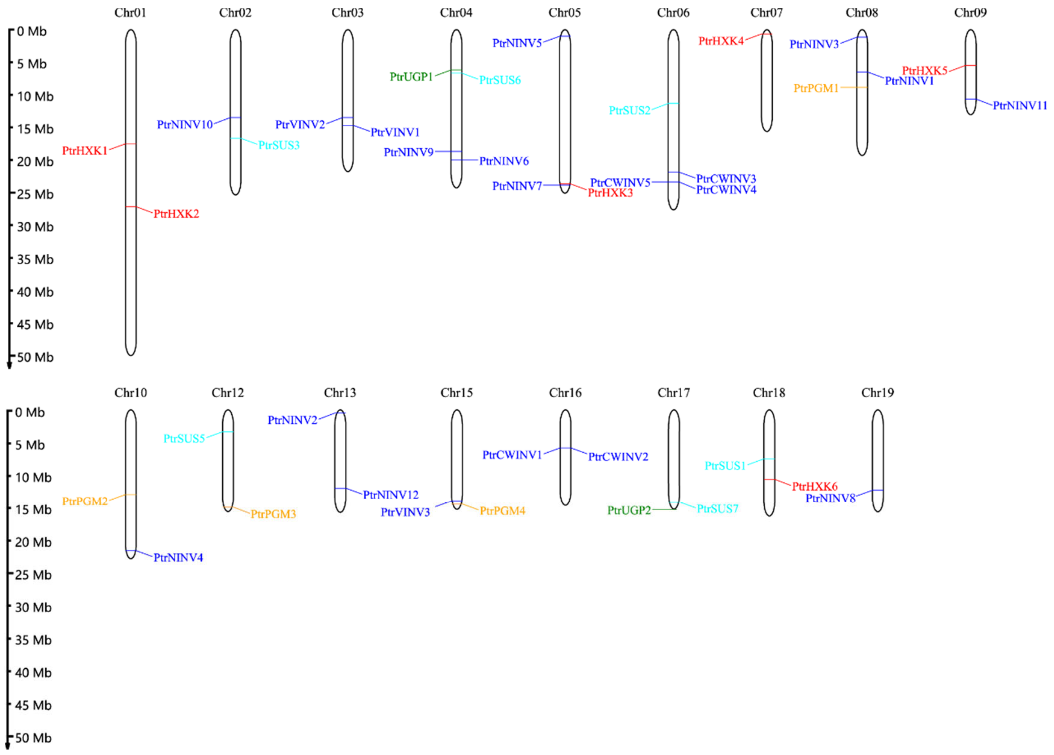

Figure S3. Chromosomal distribution of *PtrSUS, PtrINV, PtrHXK, PtrPGM* and *PtrUGP* genes. The chromosome number and the gene name (*PtrSUS*: cyan font; *PtrINV*: blue font; *PtrHXK*: red font; *PtrPGM*: yellow font; *PtrUG*P: green font) were written on the upper and two side of the oval, respectively. The oval represents the chromosome, and the leftmost scale indicates the chromosome length (Mb).
