# Supplementary material for: Structure and Expression Analysis of PtrSUS, PtrINV, PtrHXK, PtrPGM, and PtrUGP Gene Families in Populus trichocarpa Torr. and Gray
Source: Int J Mol Sci. 2023 Dec 8;24(24):17277. doi: 10.3390/ijms242417277 (PMC10743687; doi:10.3390/ijms242417277)
Supplement: Supplementary file 1 [file ijms-24-17277-s001.zip › Table S1.pdf]

Table S1. The contents of soluble sugar, reducing sugar, starch, total nitrogen, lignin, cellulose, hemicellulose, chlorophyll and height.

Name (unit)	0.1 mM NH_4NO_3 - 400 ppm CO_2	1 mM NH_4NO_3 - 400 ppm CO_2	5 mM NH_4NO_3 - 400 ppm CO_2	0.1 mM NH_4NO_3 - 800 ppm CO_2	1 mM NH_4NO_3 - 800 ppm CO_2	5 mM NH_4NO_3 - 800 ppm CO_2
Soluble sugar of roots (mg・g-1·Dw)	95.2928	57.06202	47.81216	90.49529	60.12518	66.79185
	86.50231	66.56519	45.70669	103.3396	61.81054	70.62385
	95.02551	48.58398	66.98328	92.2982	52.78462	62.95984
Soluble sugar of stems (mg・g-1·Dw)	148.4904	134.382	149.8447	112.4728	92.52046	162.9898
	139.0769	120.4392	184.2916	133.7669	87.61261	157.3421
	169.3001	117.7288	164.1139	125.2829	97.47942	189.9386
Soluble sugar of leaves (mg・g-1·Dw)	455.8988	416.9659	380.1692	510.0239	363.6811	419.8266
	433.269	397.5186	445.9789	441.4033	382.009	426.2258
	505.2934	447.2577	421.9728	501.0861	485.701	453.6302
Reducing sugar of roots (mg・g-1·Dw)	27.00715	14.25129	14.55883	27.7304	15.46949	18.88412
	26.85925	14.92386	14.1614	28.37564	14.03179	19.65743
	31.8495	16.20214	19.10677	29.67846	17.72117	18.11081
Reducing sugar of stems (mg・g-1·Dw)	25.3924	25.54899	13.21084	20.67276	13.85489	11.3245
	31.27724	25.43408	17.33356	27.95149	14.96632	11.44492
	34.52543	24.67138	15.02103	28.22228	14.03255	14.86002
Reducing sugar of leaves (mg・g-1·Dw)	143.7563	103.5793	99.47165	175.391	77.92438	108.1452
	138.69	98.12222	124.0197	164.1394	89.98347	123.9502
	172.3616	110.7465	122.9344	184.4349	108.7792	129.2774
Total nitrogen of roots (mg・g-1·Dw)	8.648649	15.83333	30.62619	7.348485	9.152941	20.55556
	6.571936	13.66397	46.19289	9.356618	5.58952	16.48551
	9.183673	15.9322	10.05338	7.375231	6.476793	14.73477
Total nitrogen of stems(mg・g-1·Dw)	7.843511	8.02521	18.08874	12.53438	10.53879	7.571702
	6.732422	7.995546	19.27711	12.35401	10.7033	6.691589
	5.793103	7.180043	16.66667	12.07156	10.14831	7.403101
Total nitrogen of leaves (mg・g-1·Dw)	13.90597	16.79389	26.24521	14.13732	8.678501	10.17476
	13.49481	11.23596	19.28166	15.01976	8.711434	8.379888
	15.34247	16.31679	23.1203	12.4	6.113139	7.870036
Lignin (%)	26.03127	23.58396	16.16044	29.8654	20.07614	12.65262
	27.25493	20.97349	18.4446	29.45751	21.05506	13.54997
	27.25493	22.3603	19.42352	24.97077	18.85248	12.97893
Cellulose (%)	36.84848	42.72727	49.39394	38.18182	44.36364	54.60606
	37.27273	42.36364	47.51515	38.48485	43.87879	55.45455
	37.39394	43.57576	49.39394	38.06061	43.75758	55.0303
	36.84848	43.33333	48.90909	38.54545	44.54545	53.39394
	36.78788	40.84848	47.81818	39.0303	43.51515	49.15152
Hemicellulos (%)	20.99579	20.78526	18.28	24.38526	20.99579	20.15368
	22.78526	20.89053	19.37474	25.08	20.78526	20.36421
	23.18526	20.76421	19.73263	23.35368	20.99579	19.94316

**Height (cm)**		24.1			23			19.9			30.9			25.4			22.6	
		28.1			20			11.6			30.1			24.9			21.5	
		17			24.3			18.3			28.2			20.1			17.2	
		23.4			15.9			15			26.3			21			13.5	
		27.3			21			18.7			30.3			28.3			15.2	
		27.4			24.7			17.5			29.4			26			20.8	
		25			20.1			24.2			31.2			20.3			16.6	
		26.4			19.8			21.4			29.6			20.5			17.1	
		17			23.2			19.3			28			20			17	
**Chlorophyll content (SPAD)**	5.30	6.40	7.30	7.70	9.20	9.90	12.60	12.90	10.10	3.30	4.90	5.10	9.60	11.80	14.50	12.40	12.70	12.70
	7.40	6.60	7.50	12.30	11.20	10.40	6.90	6.70	5.20	5.70	6.80	5.70	9.60	10.10	7.20	12.90	8.80	9.80
	7.40	10.30	8.80	8.20	10.10	12.00	11.80	11.20	11.70	7.20	7.30	7.90	8.30	7.20	9.20	11.20	8.30	13.40
	6.50	5.90	6.80	11.90	10.00	10.00	14.70	13.70	10.30	7.00	6.50	6.70	9.80	9.80	8.9	13.20	14.10	14.40
	4.20	6.30	7.30	8.60	10.00	9.80	19.00	12.20	13.90	5.60	5.40	4.10	10.00	9.40	9.10	9.30	13.60	6.20
	5.30	5.30	7.10	13.80	13.00	14.50	7.50	12.20	15.10	5.10	5.20	5.20	9.90	12.70	12.60	10.20	10.80	18.70
	8.30	5.90	10.10	11.30	10.30	11.30	12.30	11.80	13.70	6.20	7.10	7.50	11.20	9.20	11.00	13.20	14.70	13.50
	6.20	6.90	7.90	12.10	11.90	12.00	14.40	13.70	15.60	6.90	6.50	7.00	10.30	10.30	10.60	14.00	13.60	12.90
	6.50	7.40	8.30	12.40	14.00	14.00	11.50	16.20	15.30	7.60	7.70	8.30	12.30	11.20	13.20	13.2	10.3	13.4
