# Supplementary material for: Structure and Expression Analysis of PtrSUS, PtrINV, PtrHXK, PtrPGM, and PtrUGP Gene Families in Populus trichocarpa Torr. and Gray
Source: Int J Mol Sci. 2023 Dec 8;24(24):17277. doi: 10.3390/ijms242417277 (PMC10743687; doi:10.3390/ijms242417277)
Supplement: Supplementary file 1 [file ijms-24-17277-s001.zip › Table S2.pdf]

Table S2. Polypeptides parameters of genes.

Gene name	Locus name Phytozome v4.1	No of amino acid	Subunit molecular weight (Da)	Isoelectric points	Aliphatic index	GRAVY	Chromosome location	Cellular localization	NCBI ID
PtrSUS1	Potri.018G063500.1	806	92486.22	6.23	91.54	-0.284	Chr18:7500284..7506672(-)	Cytoplasm	XP_002324136.3
PtrSUS2	Potri.006G136700.3	804	92142.83	6.17	93.09	-0.253	Chr06:11257839..11265268(+)	Cytoplasm	XP_006381564.1
PtrSUS3	Potri.002G202300.1	812	92961.45	5.93	89.54	-0.260	Chr02:16614167..16621281(+)	Cytoplasm	XP_002302727.1
PtrSUS5	Potri.012G037200.1	922	104065.61	6.34	81.97	-0.355	Chr12:3341105..3345812(+)	Chloroplast	XP_024437976.1
PtrSUS6	Potri.004G081300.3	831	94383.41	8.06	87.42	-0.269	Chr04:6708544..6712842(-)	Chloroplast	XP_024454512.1
PtrSUS7	Potri.017G139100.1	840	95352.25	6.49	84.83	-0.312	Chr17:14027638..14032044(+)	Chloroplast	XP_024444461.1
PtrNINV1	Potri.008g101500.2	693	78136.98	6.03	89.13	-0.246	Chr08:6406060..6410230 (+)	Cytoplasm	XP_024462542.1
PtrNINV2	Potri.013g006600.1	669	75798.52	5.68	93.87	-0.238	Chr13:434665..439926 (+)	Chloroplast	XP_002318940.3
PtrNINV3	Potri.008g024100.2	664	74258.91	6.31	87.84	-0.234	Chr08:1212386..1217134 (-)	Chloroplast	XP_002311958.2
PtrNINV4	Potri.010g236100.3	667	74708.30	5.93	85.00	-0.223	Chr10:21640740..21645513 (+)	Chloroplast	XP_002316508.2
PtNINV5	Potri.005g010800.2	658	74942.67	5.89	89.45	-0.234	Chr05:912560..917066 (+)	Cytoplasm	XP_024457615.1
PtrNINV6	Potri.004g186500.4	619	69656.27	7.46	92.02	-0.111	Chr04:19992986..19998963 (-)	Cytoplasm	XP_006384642.2
PtrNINV7	Potri.005g239400.1	556	63488.94	6.14	82.77	-0.186	Chr05:23656472..23659080 (-)	Chloroplast	XP_002307726.2

PtNINV8	Potri.019g082000. 2	558	63346.66	6.01	83.75	-0.218	Chr19:12213512..12220476 (-)	Chloroplast	XP_002325983.1
PtrNINV9	Potri.004g167500.1	574	65425.21	6.16	85.11	-0.228	Chr04:18565795..18569556 (-)	Chloroplast	XP_002306166.1
PtrNINV10	Potri.002g173600.1	723	81153.77	5.17	87.77	-0.275	Chr02:13373002..13377749 (+)	Chloroplast	XP_002301418.2
PtrNINV11	Potri.009g129000.1	575	65669.44	5.93	84.44	-0.243	Chr09:10574593..10578253(-)	Chloroplast	XP_002312983.1
PtrNINV12	Potri.013g110800.2	558	63529.92	6.22	83.91	-0.221	Chr13:11897313..11904543 (-)	Chloroplast	XP_006376270.1
PtrCWINV1	Potri.016g077400.2	580	65397.15	7.00	78.27	-0.383	Chr16:5823103..5827107 (-)	Cell wall	PNT03998.1
PtrCWINV2	Potri.016G077500.1	589	66198.82	6.95	79.39	-0.376	Chr16:5833696..5837689 (-)	Cell wall	PNT05684.1
PtrCWINV3	Potri.006G210600.1	579	65427.42	8.60	83.11	-0.310	Chr06:21776023..21781008 (-)	Cell wall	PNT05683.1
PtrCWINV4	Potri.006g227500.1	577	65596.16	5.03	80.05	-0.299	Chr06:23165293..23168940 (-)	Cell wall	PNS95351.1
PtrCWINV5	Potri.006g227400.1	571	64770.29	8.03	77.79	-0.464	Chr06:23161445..23164400 (-)	Cell wall	XP_024443338.1
PtrVINV1	Potri.003g126300.2	649	72131.18	5.72	81.65	-0.308	Chr03:14579537..14581828 (+)	Vacuole	XP_024443362.1
PtrVINV2	Potri.003g112600.1	663	73338.68	5.81	82.90	-0.306	Chr03:13500387..13505882 (+)	Vacuole	XP_002309419.2
PtrVINV3	Potri.015g127100.1	640	71765.44	4.98	77.93	-0.339	Chr15:13873693..13878684 (+)	Vacuole	XP_024458233.1
PtrHXK1	Potri.001G190400.1	495	53626.97	5.70	97.33	-0.002	Chr01:17368682..17373498(-)	Chloroplast	XP_002299739.1
PtrHXK2	Potri.001G254800.1	509	54966.48	6.50	103.58	0.103	Chr01:27085477..27092070(-)	Chloroplast	XP_002298298.1
PtrHXK3	Potri.005G238600.1	496	53093.53	5.65	88.08	-0.036	Chr05:23614352..23619923(+)	Chloroplast	XP_024456251.1

PtrHXK4	Potri.007G009300.1	491	53565.94	6.20	89.71	-0.173	Chr07:725874..729485(-)	Chloroplast	XP_024460595.1
PtrHXK5	Potri.009G050000.1	509	54940.36	6.54	101.87	0.070	Chr09:5517300..5522735(-)	Chloroplast	XP_002313358.2
PtrHXK6	Potri.018G088300.1	499	53823.90	5.63	96.35	0.035	Chr18:10693691..10699858(-)	Chloroplast	XP_002325031.1
PtrPGM1	Potri.008G132500.3	583	63070.32	5.49	82.42	-0.177	Chr08:8753363..8763238(+)	Cytoplasm	XP_024463224.1
PtrPGM2	Potri.010G109500.2	583	63102.39	5.31	82.44	-0.166	Chr10:12870858..12880632(-)	Cytoplasm	XP_002315869.3
PtrPGM3	Potri.012G132500.1	638	69357.71	5.90	85.57	-0.151	Chr12:14909532..14918176(-)	Chloroplast	XP_024437759.1
PtrPGM4	Potri.015G134700.1	636	68838.97	5.44	83.39	-0.159	Chr15:14435535..14443548(-)	Chloroplast	XP_024441768.1
PtrUGP1	Potri.004G074400.5	470	51757.41	5.41	101.96	-0.150	Chr04:6214132..6219846(+)	Cytoplasm	XP_006384119.1
PtrUGP2	Potri.017G144700.1	470	51605.38	5.78	103.43	-0.134	Chr17:15254830..15260627(-)	Cytoplasm	XP_006372413.1
