# Supplementary material for: Structure and Expression Analysis of PtrSUS, PtrINV, PtrHXK, PtrPGM, and PtrUGP Gene Families in Populus trichocarpa Torr. and Gray
Source: Int J Mol Sci. 2023 Dec 8;24(24):17277. doi: 10.3390/ijms242417277 (PMC10743687; doi:10.3390/ijms242417277)
Supplement: Supplementary file 1 [file ijms-24-17277-s001.zip › Table S3.pdf]

Table S3. Motif sequences of SUS proteins identified in *P. trichocarpa* by MEME tools.

Motif	Width	P-value	Best possible match
1	50	3.7e-62	RNGELYRYIADTKGAFVQPALYEAFGLTVIEAMTCGLPTFATNQGGPAEI
2	50	1.5e-62	YHFSCQFTADJIAMNHTDFIITSTYQEIAGSKDTVGQYESHTAFTLPGLY
3	50	1.6e-57	VFNVVIFSPHGYFGQABVLGLPDTGGQVVYILDQVRALEEELLLRIKQQG
4	50	4.9e-60	LQGKPDLIIGNYSDGNLVASLLAHKLGITQCTIAHALEKTKYPDSDIKWK
5	50	3.1e-54	GFHIDPYNGDZAAEJJADFFEKCKVDPGYWBKISAGGLQRINECYTWKIY
6	50	6.7e-56	EHIGYLEDKKKPIIFSMARLDTVKNLTGLVEWYGKNTKLRELVNLVVVGG
7	50	8.7e-54	RVVHGIBVFDPKFNIVSPGADQSIYFPYTEKQKRLTSFHPEIEELLYSSV
8	50	2.0e-53	TRLIPDARGTTCNQELERIYGTEHSNILRVPFRDEKGVLRKWISRFDVWP
9	50	5.3e-50	QEAIVLPPYVALAIRPRPGVWEYVKVNVQDLSVEGJTVSEYLKFKEEJVD
10	50	2.6e-49	DTPYQNFEHKFQEIGFERGWGDTAERVKETMRLLLDVLQAPDPMTLETFL

Motif sequences of NINV proteins identified in *P. trichocarpa* by MEME tools.

Motif	Width	P-value	Best possible match
1	41	9.7e-47	HALSFHIREYYWIDLKQLNEIYRYKTEEYSHTAVNKFNIYP
2	50	8.8e-59	KICYPAJESHEWRIVTGSDPKNTRWSYHNGGSWPVLLWLLTAACIKTGRP
3	50	2.1e-54	ADFGESAIGRVAPVDSGFWWIILLRAYTKSTGDLSLAERPEVQTGMKLIL
4	50	3.8e-61	LCLSDGFDTFPTLLCADGCCMIDRRMGIYGYPJEIQALFYSALRCAKEML
5	50	8.1e-49	WLFDFMPNRGGYLIGNVSPARMDFRWFTLGNCWAILSSLATPEQSTAIMD
6	50	9.8e-46	EYYDGKLGRFIGKQARKFQTWSIAGYLVAKMLLEDPSHLGMJSLEEDKZL
7	30	1.8e-35	EPEIVKNFLLKTLQLQSWEKTIDCFSLGZG
8	29	4.1e-30	DPNSEEVLNYDQVFVRDFVPSALAFLMNG
9	33	3.5e-27	NGFETHPMVEEAWELLRRSLVYFRGQPVGTIAA
10	15	8.0e-17	LIEARWEELVGEMPL

The domains were added with a background color to make it easier to identify.

Motif sequences of CWINV and VINV proteins identified in P. trichocarpa by MEME tools.

Motif	Width	P-value	Best possible match
1	50	1.0e-55	LRYDYGNFYASKTFFDPSTNRRILWGWVNESDSEQDDVDKGWAGJQSIPR
2	50	2.0e-58	WAHSVSKDLINWEHLEPAJYPSKWFDINGCWSGSATILPBGEPVILYTGI
3	50	3.0e-50	TDEKLSLRSLIDHSIVESFGAGGRTVITSRVYPTIAIYEKARLFVFNNGT
4	50	1.6e-60	PLHSVPGTGMWECPDFYPVSLSGENGLDTSVNGPNVKHVLKASLDDTRYE
5	49	1.0e-51	NNVQVQNLAVPANLSDPLLREWVKYDDNPIVTPPPGVNGSAFRDPTTAW
6	50	7.8e-52	GKQLLQWPVEEIEKLRGKNVQFSNQKLKQGSVVEVEGITAAQADVDVTFE
7	29	5.4e-29	MNDPNGPLYYKGWYHLFYQYNPKGAVWGB
8	42	2.1e-44	VCAQKGAKARGGLGPFGLLTLASEBLEEFTPVFFRVFKANBG
9	15	1.9e-19	QWHRTGYHFQPPKNW
10	21	1.6e-21	DGKWRILIGSKRNNTGIAYLY

The domains were added with a background color to make it easier to identify.

Motif sequences of HXK proteins identified in *P. trichocarpa* by MEME tools.

Motif	Width	P-value	Best possible match
1	37	9.6e-42	LPLTEYDIDLDSESPNPGEQIFEKLISGMYLGEIVRR
2	50	9.7e-52	DMRVSALVNDTVGTLAGGRYHDADVVAAVILGTGTNAAYVERADAIPKWQ
3	50	7.2e-61	MHAGLASEGGSKLKMLJSYVDNLPSGDEKGLYYALDLGGTNFRVLRVQLG
4	50	5.5e-45	FIASTLAQFVEKEEEGFEPSPGRQRELGFTFSFPVKQTSIASGILIKWTK
5	39	2.3e-40	VPPKLSIPFILRTPDLAAMHQDDSPDLKVVGKKLKEILE
6	39	3.3e-36	EMKKTVVAMDGGLYEHYSMFRKYLHEALNELLGEEVSKN
7	41	1.5e-41	ISNVSLKVRKVVVEVCDIVATRGARLAAAGIVGIJKKIGRD
8	21	2.8e-25	LLPTSGEMVINMEWGNFRSSH
9	29	6.6e-27	WKRVVRILRELZEACGTPIGKLRQVADAM
10	29	1.3e-28	RDGGIVSQEFEEVSIPPHLMTGTSEELFD

The domains were added with a background color to make it easier to identify.

Motif sequences of PGM proteins identified in *P. trichocarpa* by MEME tools.

Motif	Width	P-value	Best possible match
1	50	7.6e-64	NLPFFEVPTGWKFFGNLMDAGKLSICGEESFGTGSDHIREKDGIWAVLAW
2	50	1.1e-58	FCYDALHGVTGAYAKPIFVDELGAQEDSJSNGVPKEDFGGGHPDPNLTYA
3	50	4.8e-58	FILSASHNPGGPEYDWGIKYNYENGGPAPEGITDKIYGNTKSIKEYKTAD
4	50	6.9e-58	KQGIRYLFEDGSRJIFRLSGTGSEGATIRJYIEQYEPDPSKHGRDAQDAL
5	50	8.9e-56	QKPGTSGLRKKVKVFKZPNYLANWIQALFNALPPZDYKGGVLVLGGDGRY
6	50	1.4e-54	YKNKDKKPGGKLVSVEDIVKEHWATYGRNYYSRYDYEEVDAGGAKELIQY
7	41	6.9e-49	NYEEEPPDFGAASDGDGDRNMILGKGFFVTPSDSVAIIAAN
8	41	3.0e-45	FNKDAIQIIIKIAAGNGVGKIWVGKEGJLSTPAVSAVIRER
9	38	4.6e-41	YGNFDVEVFDPVSDYLELMENIFDFELIKKLLSRPDFR
10	29	3.4e-33	VEAIPYFSAGPKGLARSMPTSGALDRVAE

The domains were added with a background color to make it easier to identify.

Motif sequences of UGP proteins identified in *P. trichocarpa* by MEME tools.

Motif	Width	P-value	Best possible match
1	50	1.7e-65	EKYSNSNIEIHTFNQSQYPRLVVDDFVPLPSKGHTDKDGWYPPGHGDVFP
2	50	1.5e-63	TTMGCTGPKSVIEVRNGLTFLDLIVIQIENLNKKYGCSVPLLLMNSFNTH
3	50	3.7e-64	NHLIRNKNEYCMEVTPKTLADVKGGTLISYEGKVQLLEIAQVPDQHVNEF
4	50	1.8e-62	AAIKFFDHAIGINVPRSRFLPVKASSDLLLVQSDLYTLVDGFVIRNPART
5	50	6.5e-63	MATDTEKISQLKSAVANLNQISESEKTGFVNLVSRYLSGEAQQVEWSKIQ
6	50	2.5e-62	KFKIFNTNNLWVNLKAIKRLVEADALEMEIIPNPKEVDGVKVLQLETAAG
7	50	4.7e-61	PANPSIELGPEFKKVANFLSRFKSIPSIIELDSLKVVGDVWFGAGITLKG
8	29	6.4e-36	TPTDEVVVPYDTLEPTPEEPEETKKLLDK
9	29	9.6e-36	NSGKLDALLSKGKEYVFVANSDNLGAVVD
10	29	5.3e-34	VSIVVKSGVKLEIPEGVILZNKEINGPED

The domains were added with a background color to make it easier to identify.
