# Supplementary material for: Structure and Expression Analysis of PtrSUS, PtrINV, PtrHXK, PtrPGM, and PtrUGP Gene Families in Populus trichocarpa Torr. and Gray
Source: Int J Mol Sci. 2023 Dec 8;24(24):17277. doi: 10.3390/ijms242417277 (PMC10743687; doi:10.3390/ijms242417277)
Supplement: Supplementary file 1 [file ijms-24-17277-s001.zip › Table S4.pdf]

Table S4. The secondary structures prediction of PtrSUSs, PtrINVs, PtrHXKs, PtrPGMs and PtrUGPs based on the sequence using NPSA

	Alpha helix (Hh)	Extended strand (Ee)	Beta turn (Tt)	Random coil (Cc)
PtrSUS1	53.79%	13.17%	6.58%	26.46%
PtrSUS2	53.18%	13.33%	6.60%	26.90%
PtrSUS3	53.76%	12.82%	7.52%	25.89%
PtrSUS5	49.29%	11.51%	6.08%	33.12%
PtrSUS6	54.13%	12.57%	6.83%	26.47%
PtrSUS7	51.73%	12.04%	6.91%	29.32%
PtrNINV1	36.96%	17.44%	5.81%	39.79%
PtrNINV2	34.13%	18.26%	5.39%	42.22%
PtrNINV3	38.61%	14.03%	6.49%	40.87%
PtrNINV4	38.61%	14.03%	6.49%	40.87%
PtNINV5	37.99%	16.22%	5.86%	39.94%
PtrNINV6	40.29%	14.72%	7.28%	37.70%
PtrNINV7	43.06%	14.95%	7.39%	34.59%
PtNINV8	41.29%	14.72%	6.28%	37.70%
PtrNINV9	42.41%	13.61%	6.28%	37.70%
PtrNINV10	38.92%	13.99%	4.57%	42.52%
PtrNINV11	43.73%	13.59%	6.62%	36.06%
PtrNINV12	40.93%	14.90%	6.28%	37.88%
PtrCWINV1	20.03%	24.35%	5.53%	50.09%
PtrCWINV2	15.37%	24.56%	6.18%	53.89%
PtrCWINV3	16.26%	25.43%	5.71%	52.60%
PtrCWINV4	19.10%	25.35%	5.90%	5.90%
PtrCWINV5	18.77%	24.04%	5.96%	51.23%
PtrVINV1	13.67%	27.72%	6.55%	52.06%
PtrVINV2	16.16%	22.66%	5.89%	55.29%
PtrVINV3	15.34%	23.00%	5.63%	56.03%
PtrHXK1	47.17%	13.77%	6.07%	33.00%
PtrHXK2	43.70%	14.37%	5.12%	36.81%
PtrHXK3	41.01%	15.35%	7.88%	35.76%
PtrHXK4	47.81%	14.14%	4.58%	33.47%
PtrHXK5	46.65%	12.99%	4.92%	35.43%
PtrHXK6	46.99%	13.05%	6.22%	33.73%
PtrPGM1	35.63%	16.58%	6.90%	40.89%
PtrPGM2	34.71%	16.67%	7.73%	40.89%
PtrPGM3	35.79%	17.27%	7.54%	39.40%
PtrPGM4	33.39%	17.80%	7.40%	41.42%
PtrUGP1	33.05%	20.68%	7.46%	38.81%
PtrUGP2	34.33%	20.26%	6.82%	38.59%
