# Supplementary material for: Structure and Expression Analysis of PtrSUS, PtrINV, PtrHXK, PtrPGM, and PtrUGP Gene Families in Populus trichocarpa Torr. and Gray
Source: Int J Mol Sci. 2023 Dec 8;24(24):17277. doi: 10.3390/ijms242417277 (PMC10743687; doi:10.3390/ijms242417277)
Supplement: Supplementary file 1 [file ijms-24-17277-s001.zip › Table S5.pdf]

Table S5. The template information for the tertiary structure prediction PtrSUSs, PtrINVs, PtrHXKs, PtrPGMs and PtrUGPs

Protein	Template	GMQE	Oligo-State	Seq Identity	Seq Similarity	Description
PtrSUS1	3s27.1.A	0.90	Homo-tetramer	83.69%	0.57	Sucrose synthase 1
PtrSUS2	3s27.1.A	0.90	Homo-tetramer	84.23%	0.57	Sucrose synthase 1
PtrSUS3	3s27.1.A	0.80	Homo-tetramer	67.74%	0.51	Sucrose synthase 1
PtrSUS5	4rbn.1.A	0.63	Homo-tetramer	45.63%	0.42	Sucrose synthase: Glycosyl transferases group 1
PtrSUS6	4rbn.1.A	0.69	Homo-tetramer	44.22%	0.42	Sucrose synthase: Glycosyl transferases group 1
PtrSUS7	3s27.1.A	0.80	Homo-tetramer	54.99%	0.46	Sucrose synthase 1
PtrNINV1	5gor.1.A	0.55	Homo-hexamer	55.38%	0.48	Alkaline Invertase
PtrNINV2	6ttj.1.A	0.55	Homo-hexamer	57.48%	0.48	Alkaline/neutral invertase CINV1
PtrNINV3	5gor.1.A	0.55	Homo-hexamer	54.82%	0.47	Alkaline Invertase
PtrNINV4	5gor.1.A	0.55	Homo-hexamer	54.82%	0.47	Alkaline Invertase
PtNINV5	5gor.1.A	0.55	Homo-hexamer	53.71%	0.47	Alkaline Invertase
PtrNINV6	6ttj.1.A	0.60	Homo-hexamer	61.30%	0.49	Alkaline/neutral invertase CINV1
PtrNINV7	6ttj.1.A	0.79	Homo-hexamer	75.52%	0.54	Alkaline/neutral invertase CINV1
PtNINV8	6ttj.1.A	0.81	Homo-hexamer	82.75%	0.57	Alkaline/neutral invertase CINV1
PtrNINV9	6ttj.1.A	0.78	Homo-hexamer	76.81%	0.55	Alkaline/neutral invertase CINV1
PtrNINV10	6ttj.1.A	0.53	Homo-hexamer	71.76%	0.53	Alkaline/neutral invertase CINV1
PtrNINV11	6ttj.1.A	0.78	Homo-hexamer	75.23%	0.55	Alkaline/neutral invertase CINV1
PtrNINV12	6ttj.1.A	0.81	Homo-hexamer	83.09%	0.57	Alkaline/neutral invertase CINV1
PtrCWINV1	2xqr.1.A	0.78	Monomer	58.93%	0.49	β-fructofuranosidase,insoluble isoenzyme CWINV1
PtrCWINV2	2xqr.1.A	0.77	Monomer	57.49%	0.48	β-fructofuranosidase,insoluble isoenzyme CWINV1
PtrCWINV3	2xqr.1.A	0.78	Monomer	59.81%	0.49	β-fructofuranosidase,insoluble isoenzyme CWINV1
PtrCWINV4	1st8.1.A	0.82	Monomer	59.10%	0.49	fructan 1-exohydrolase IIa

PtrCWINV5	1st8.1.A	0.82	Monomer	60.26%	0.50	fructan 1-exohydrolase IIa
PtrVINV1	3ugf.1.A	0.88	Monomer	64.27%	0.51	Sucrose:(Sucrose/fructan) 6-fructosyltransferase
PtrVINV2	3ugf.1.A	0.74	Monomer	65.55%	0.52	Sucrose:(Sucrose/fructan) 6-fructosyltransferase
PtrVINV3	3ugf.1.A	0.76	Monomer	64.23%	0.51	Sucrose:(Sucrose/fructan) 6-fructosyltransferase
PtrHXK1	6jj8.3.A	0.84	Monomer	65.73%	0.50	Rice hexokinase 6
PtrHXK2	4qs8.1.A	0.77	Monomer	57.67%	0.46	Hexokinase-1
PtrHXK3	6jj8.3.A	0.81	Monomer	56.33%	0.46	Rice hexokinase 6
PtrHXK4	6jj8.3.A	0.75	Monomer	47.90%	0.44	Rice hexokinase 6
PtrHXK5	4qs8.1.A	0.76	Monomer	56.68%	0.46	Hexokinase-1
PtrHXK6	4qs8.1.A	0.85	Monomer	80.04%	0.55	Hexokinase-1
PtrPGM1	7p5o.1.A	0.73	Monomer	54.00%	0.45	Phosphoglucomutase
PtrPGM2	5vg7.1.A	0.79	Monomer	57.55%	0.47	Phosphoglucomutase-1
PtrPGM3	5vg7.1.A	0.69	Monomer	57.88%	0.47	Phosphoglucomutase-1
PtrPGM4	5epc.1.A	0.70	Monomer	55.41%	0.46	Phosphoglucomutase-1
PtrUGP1	2icx.1.A	0.93	Monomer	83.55%	0.56	Probable UTP-glucose-1-phosphate uridylyltransferase 2
PtrUGP2	2icx.1.A	0.93	Monomer	83.08%	0.56	Probable UTP-glucose-1-phosphate uridylyltransferase 2
