# Supplementary material for: Structure and Expression Analysis of PtrSUS, PtrINV, PtrHXK, PtrPGM, and PtrUGP Gene Families in Populus trichocarpa Torr. and Gray
Source: Int J Mol Sci. 2023 Dec 8;24(24):17277. doi: 10.3390/ijms242417277 (PMC10743687; doi:10.3390/ijms242417277)
Supplement: Supplementary file 1 [file ijms-24-17277-s001.zip › Table S6.pdf]

Table S6. Gene expression levels in different tissue and under different treatments.

In different tissue sites															
**Gene**	**Roots**			**Upper stems**			**Lower stems**			**Young leaves**			**Mature leaves**		
PtrSUS1	0.003333	0.140531	-0.00667	2.16333	2.17333	2.25333	0.016663	0.146663	0.266663	-5.49667	-4.78667	-4.95667	-6.59334	-6.56334	-6.34334
PtrSUS2	0.046667	0.07667	-0.12333	2.26667	2.13667	2.10667	0.160003	0.230003	0.220003	-4.05333	-4.14333	-4.12333	-5.08	-5.12	-5.24
PtrSUS3	0.006667	0.00667	-0.01333	1.30667	1.51667	1.39667	-0.29	-0.08	-0.06	-0.35333	-0.38333	-0.32333	1.990003	2.080003	1.960003
PtrSUS5	-0.25333	-0.0333	0.2867	2.6467	2.6967	1.9767	0.240033	-0.05997	-0.12997	-0.4933	-0.3733	-0.4933	-2.92997	-3.37997	-3.51997
PtrSUS6	-0.08333	-0.01333	0.09667	2.67667	2.83667	3.02667	0.880003	0.740003	0.680003	0.45667	0.65667	0.64667	1.620003	1.450003	1.550003
PtrSUS7	0.096667	-0.11333	0.01667	1.90667	1.82667	1.74667	-1.13	-0.77	-0.86	-1.07666	-0.85666	-1.28666	-3.69	-5	-4.58
PtrNINV1	0.133564	-0.03653	-0.09696	1.06695	1.076559	0.796598	0.136191	0.986957	0.80653	-0.59946	-0.02034	-0.45997	0.186501	-0.66429	-0.11404
PtrNINV2	-0.03653	0.073135	-0.03653	0.286881	0.107018	0.276199	-1.02915	-0.97994	-0.76121	1.233275	1.313246	1.123004	1.396708	1.376846	1.016496
PtrNINV3	-0.03653	-0.05739	0.09356	0.609755	0.60027	0.83996	0.62293	0.343692	1.013641	2.913416	2.603359	2.953265	1.560226	1.479748	1.509949
PtrNINV4	-0.05289	-0.16327	0.21661	0.97306	0.97306	0.853597	1.006478	0.836732	0.656268	2.21661	2.196607	2.186817	2.533314	2.493391	2.513238
PtrNINV5	-0.24982	0.089498	0.159629	-0.26361	0.04684	-0.01304	-0.01013	-0.10005	-0.10005	0.85997	1.289834	1.309758	1.21661	1.316725	1.186501
PtrNINV6	-0.05739	-0.06643	0.123004	-0.77596	-0.72738	-0.39593	0.983313	0.803227	0.822934	-0.81097	-0.1504	-0.52907	1.219711	1.390117	1.289834
PtrNINV7	-0.12973	0.22033	-0.08927	0.950095	0.350497	0.919912	2.14991	1.799916	2.049979	1.906506	1.80653	1.726831	-0.04394	0.736388	1.186501
PtrNINV8	-0.0862	-0.11716	0.202888	-0.85726	-0.66658	-0.69666	0.1137	-0.31653	0.233888	-1.46993	-1.1488	-1.08009	-1.85204	-2.04097	-1.80088
PtrNINV9	0.070389	0.070389	-0.13924	0.279768	0.290424	0.159629	0.70044	0.649845	0.789938	-2.81604	-2.8997	-2.06492	-3.2109	-2.92139	-2.93236

PtrNINV10	0.029983	-0.18935	0.159629	-0.74662	-0.77596	-0.0862	-0.5043	-1.30401	-0.92413	2.590003	2.480007	2.760008	-0.74662	-1.26534	-0.79586
PtrNINV11	-0.05289	-0.29336	0.347099	0.87342	1.053111	0.822934	0.082703	0.183963	0.822934	-2.14561	-1.32193	-1.22432	-3.53952	-3.32193	-4.71786
PtrNINV12	-0.17951	-0.06945	0.249749	0.210389	0.049631	0.140124	2.703322	2.603359	2.763412	-1.86775	-1.41889	-1.78091	-1.23447	-1.24469	-1.16488
PtrCWINV1	-0.01667	-0.2367	0.2533	-1.42003	-0.84003	-1.47003	-6.50003	-5.51003	-5.20003	0.0033	-0.1767	0.0933	-1.42003	-0.84003	-1.47003
PtrCWINV2	0.386667	-0.11333	-0.27333	-0.69333	-0.83333	-1.18333	-0.19333	0.416667	0.316667	-1.19667	-1.68667	-1.24667	-2.12	-2.24	-2.13
PtrCWINV3	-0.31667	-0.17	-0.18	-3.23	-2.42	-1.36	-0.09667	-0.00667	-0.12667	-1.92667	-1.85667	-1.46667	1.35333	1.46333	1.45333
PtrCWINV4	0.01	0.35333	0.30333	-1.53334	-1.92334	-1.80334	-0.92667	-0.54667	-0.34667	-3.12334	-2.58334	-3.31334	-0.67	-0.36	-0.44
PtrCWINV5	-0.28	-0.12	0.4	-5.81667	-5.19667	-5.81667	-7.11	-6.34	-6.97	-6.78667	-7.52667	-6.92667	-8.12333	-6.90333	-8.68333
PtrVINV1	-0.11333	0.63	0.15	0.743333	1.873333	1.023333	0.146667	0.786667	0.566667	4.23	4.44	4.07	0.033333	1.183333	0.613333
PtrVINV2	0.17333	0.00333	-0.03	-0.07	0.039997	-0.81667	-0.75667	-0.91667	-1.64667	-1.54667	-1.68667	-1.03667	-1.03667	-1.31667	-2.29667
PtrVINV3	-0.25333	0.426667	-0.17333	-0.35	0.27	-0.39	-0.8	-0.07	0.13	0.223333	0.343333	0.453333	-2.05	-1.1	-1.77
PtrHXK1	0.1	-0.06	-0.04	1.95	2	1.89	0.493333	0.433333	0.473333	0.67	0.94	0.43	-0.88667	-0.67667	-0.61667
PtrHXK2	-0.11	-0.11	0.22	1.81	1.8	2.26	-0.25667	0.143333	0.123333	0.6	0.59	0.58	0.703333	1.503333	1.203333
PtrHXK3	-0.14667	0.01333	0.13333	4.25333	4.29333	4.30333	2.436663	2.466663	2.496663	1.87333	1.80333	1.89333	0.416663	0.246663	0.366663
PtrHXK4	0.236667	-0.03337	-0.20337	1.1033	1.3333	1.3433	1.766633	1.476633	2.016633	2.5733	2.6933	2.9133	0.726633	0.816633	0.936633
PtrHXK5	0.26	-0.09	-0.17	2.18	2.13	2.21	-0.28667	-0.20667	-0.26667	1.51	1.78	1.54	0.963333	0.713333	0.973333
PtrHXK6	-0.11333	0.03667	0.07667	0.36667	0.70667	0.39667	-1.03	-0.98	-0.95	0.62667	0.72667	0.78667	1.480003	1.520003	1.460003
PtrPGM1	0.023333	0.06333	-0.08667	2.87333	2.96333	3.03333	0.396663	0.206663	-0.16334	1.83333	1.91333	2.15333	2.616663	2.536663	2.496663
PtrPGM2	-0.09	0.22	-0.13	1.96	2	1.91	0.523333	0.193333	0.513333	1.8	1.72	1.72	1.393333	1.313333	1.123333

PtrPGM3	-0.04667	0.05333	-0.00667	2.85333	2.83333	2.69333	1.296663	1.466663	1.376663	3.34333	3.41333	3.18333	3.416663	3.386663	3.396663
PtrPGM4	0.02	0.11	-0.13	3.09	2.98	3.1	0.693333	0.703333	1.023333	3.69	3.47	3.54	2.583333	2.683333	3.043333
PtrUGP1	0.02	0.11	-0.13	3.09	2.98	3.1	0.693333	0.703333	1.023333	3.69	3.47	3.54	2.583333	2.683333	3.043333
PtrUGP2	0.116667	-0.07333	-0.04333	2.18667	2.28667	2.17667	-0.2	-0.41	-0.53	-1.22666	-1.10666	-1.10666	-0.55	-0.57	-0.59

In different treatment

Gene	0.1 mM NH_4NO_3 - 400 ppm CO_2			1 mM NH_4NO_3 - 400 ppm CO_2			5 mM NH_4NO_3 - 400 ppm CO_2			0.1 mM NH_4NO_3 - 800 ppm CO_2			1 mM NH_4NO_3 - 800 ppm CO_2			5 mM NH_4NO_3 - 800 ppm CO_2		
	**b**			**e**			**a**			**c**			**d**			**b**		
PtrSUS1	-2.48	-1.73	- 1.72	-0.02	-0.02	0.05	- 3.06	-3.04	-2.97	-1.06	-1.00	-1.09	- 0.34	- 0.38	-0.33	-2.10	-2.00	-2.01
	**c**			**d**			**a**			**e**			**c**			**b**		
PtrSUS2	-0.43	-0.09	- 0.24	0.04	0.04	-0.09	- 0.82	-0.87	-0.85	0.38	0.29	0.30	- 0.18	- 0.21	-0.36	-0.49	-0.54	-0.56
	**b**			**c**			**e**			**a**			**e**			**d**		
PtrSUS3	-0.13	-0.42	- 0.65	-0.04	0.04	0.01	1.47	1.48	1.52	-0.83	-0.71	-0.88	1.73	1.66	1.67	0.52	0.48	0.68
	**a**			**b**			**c**			**b**			**d**			**c**		
PtrSUS5	-0.40	-0.21	- 0.62	0.07	-0.08	0.00	0.33	0.43	0.58	0.22	0.03	0.08	1.23	1.26	1.06	0.50	0.51	0.39
	**a**			**bc**			**c**			**ab**			**d**			**ab**		
PtrSUS6	-0.68	-0.41	- 0.58	-0.42	0.16	0.26	0.12	0.22	0.07	-0.25	-0.21	-0.33	1.10	1.05	0.99	-0.24	-0.31	-0.37

		a			b			b			ab			c			ab	
PtrSUS7	-0.36	-0.21	- 0.47	0.06	0.18	-0.23	0.24	0.25	-0.11	-0.18	0.08	-0.18	0.88	0.75	0.61	-0.06	-0.12	-0.02
		b			c			c			a			c			d	
PtrNINV1	-0.21	-0.34	- 0.57	-0.02	0.07	-0.05	- 0.01	0.06	0.00	-0.47	-0.56	-0.60	0.71	0.68	0.81	0.19	0.23	0.21
		bc			ab			cd			a			bc			d	
PtrNINV2	0.36	-0.04	0.14	-0.19	-0.23	0.41	0.40	0.88	0.52	-0.47	-0.61	-0.03	0.19	0.40	0.65	0.83	1.16	1.16
		a			d			cd			b			e			c	
PtrNINV3	-0.99	-1.28	- 1.01	-0.03	-0.08	0.11	0.08	-0.02	-0.20	-0.73	-0.74	-0.84	0.44	0.34	0.31	-0.19	-0.36	-0.15
		a			bc			b			b			c			d	
PtrNINV4	-1.22	-0.79	- 0.87	0.24	-0.16	-0.08	- 0.41	-0.19	-0.25	-0.30	-0.11	-0.41	0.27	0.19	0.17	-0.13	-0.22	-0.31
		a			a			c			a			b			b	
PtNINV5	0.30	0.23	0.05	0.01	-0.02	0.01	0.88	0.93	1.01	0.24	0.16	0.06	0.57	0.73	0.61	0.25	0.60	0.53
		a			bc			d			b			e			cd	
PtrNINV6	-0.54	-0.70	- 0.74	-0.11	0.09	0.02	0.15	0.20	0.38	-0.28	-0.12	-0.06	0.86	0.87	1.00	0.10	0.22	0.17
		ab			c			b			ab			a			ab	
PtrNINV7	-1.22	-1.48	- 0.66	-0.01	0.02	-0.01	- 1.01	-0.19	-0.65	-0.82	-1.00	-0.95	- 1.25	- 0.94	-1.51	-0.75	-0.91	-0.36

		**a**			**d**			**a**			**b**			**e**			**c**	
PtNINV8																		
			-				-											
	-0.76	-0.94	0.95	0.10	0.07	-0.16	0.87	-0.90	-0.94	-0.51	-0.43	-0.52	0.25	0.29	0.31	-0.25	-0.27	-0.22
		**b**			**d**			**a**			**c**			**e**			**b**	
PtrNINV9																		
			-				-											
	-0.67	-0.49	0.60	0.00	0.22	0.24	0.97	-1.07	-0.92	-0.28	-0.35	-0.37	0.57	0.57	0.61	-0.73	-0.67	-0.44
		**de**			**e**			**a**			**cd**			**b**			**bc**	
PtrNINV10																		
			-				-						-	-				
	-0.89	-0.57	0.26	0.00	-0.37	0.38	3.28	-3.27	-3.76	-1.37	-1.55	-1.20	2.54	1.51	-3.41	-2.07	-2.33	-1.98
		**a**			**d**			**b**			**bc**			**bcd**			**cd**	
PtrNINV11																		
			-				-						-	-				
	-3.52	-4.10	6.77	0.18	-0.10	-0.08	2.65	-2.50	-2.30	-1.56	-1.66	-1.46	1.62	0.96	-1.36	-0.62	-0.88	-0.94
		**a**			**b**			**c**			**b**			**d**			**c**	
PtrNINV12																		
			-															
	-0.55	-0.34	0.43	0.04	-0.06	0.03	0.43	0.46	0.46	0.08	0.10	0.08	0.69	0.68	0.77	0.46	0.42	0.36
		**d**			**d**			**a**			**d**			**c**			**b**	
PtrVINV1																		
			-				-						-	-				
	-0.15	-0.01	0.30	-0.27	0.47	-0.21	4.77	-5.19	-5.19	-0.40	-0.25	0.02	1.16	1.89	-1.87	-2.74	-2.90	-3.11
		**a**			**d**			**f**			**c**			**b**			**e**	
PtrVINV2																		
			-										-	-				
	-2.33	-2.12	2.06	-0.05	-0.01	0.05	1.60	1.69	1.60	-0.19	-0.13	-0.18	0.50	0.47	-0.48	1.04	1.00	1.07
PtrVINV3		**b**			**c**			**a**			**d**			**b**			**c**	

	-0.41	-1.16	-0.94	0.05	0.03	-0.09	-3.31	-2.93	-3.55	0.73	0.41	0.92	-0.57	-0.83	-0.89	-0.11	0.10	0.04
		b			d			ab			ab			a			c	
PtrCWINV1	-2.92	-2.61	-2.80	0.40	-0.20	-0.20	-2.88	-2.95	-2.95	-3.07	-2.97	-3.05	-3.09	-2.99	-3.25	-1.89	-1.69	-1.90
		d			a			c			e			f			b	
PtrCWINV2	2.85	2.92	2.84	0.13	0.07	-0.20	0.88	1.04	0.76	3.78	3.66	3.29	3.96	3.98	4.12	0.60	0.47	0.31
		a			c			e			b			e			d	
PtrCWINV3	-1.39	-1.49	-1.79	0.19	-0.10	-0.08	1.73	1.69	1.66	-0.29	-0.34	-0.53	1.64	1.52	1.52	0.74	0.62	0.64
		a			de			b			bc			e			cd	
PtrCWINV4	-1.06	-1.24	-1.68	-0.02	0.14	-0.13	-0.92	-1.04	-0.86	-0.28	-0.75	-1.00	0.43	0.37	0.32	-0.35	-0.26	-0.46
		a			d			c			b			bc			c	
PtrCWINV5	-2.91	-3.24	-2.99	0.08	0.07	-0.16	-0.72	-1.21	-1.26	-1.53	-1.83	-2.23	-1.64	-1.35	-1.39	-1.19	-1.29	-1.52
		a			c			a			b			c			b	
PtrHXK1	-0.84	-1.09	-1.42	0.07	0.01	-0.07	-0.80	-0.78	-1.08	-0.13	-0.26	-0.56	0.19	0.16	0.14	-0.28	-0.29	-0.38
		ab			ab			a			a			c			b	
PtrHXK2	-0.24	-0.31	0.22	-0.20	-0.04	0.25	-0.42	0.19	-0.51	-0.27	-0.31	-0.35	0.71	1.14	1.07	0.28	0.27	0.19

		<u>**a**</u>			<u>**ab**</u>			<u>**ab**</u>			<u>**a**</u>			<u>**c**</u>			<u>**d**</u>	
PtrHXK3	-0.27	-0.05	-0.30	-0.07	0.00	0.08	0.00	-0.20	-0.04	-0.18	-0.11	-0.12	0.52	0.75	0.41	0.03	0.16	0.15
		<u>**b**</u>			<u>**b**</u>			<u>**c**</u>			<u>**b**</u>			<u>**a**</u>			<u>**a**</u>	
PtrHXK4	-0.28	-0.16	0.05	0.19	-0.23	0.04	0.65	0.80	0.62	0.17	-0.27	0.15	-1.16	-0.81	-1.05	-0.42	-1.46	-0.82
		<u>**a**</u>			<u>**a**</u>			<u>**b**</u>			<u>**a**</u>			<u>**b**</u>			<u>**b**</u>	
PtrHXK5	0.05	0.01	-0.17	0.15	-0.01	-0.14	0.37	0.29	0.36	0.00	-0.25	-0.33	0.54	0.36	0.18	0.69	0.53	0.37
		<u>**a**</u>			<u>**c**</u>			<u>**d**</u>			<u>**b**</u>			<u>**e**</u>			<u>**c**</u>	
PtrHXK6	-0.76	-0.84	-0.70	-0.15	0.09	0.06	0.19	0.28	0.26	-0.54	-0.59	-0.51	0.90	0.97	1.06	0.05	0.10	0.08
		<u>**a**</u>			<u>**a**</u>			<u>**bc**</u>			<u>**a**</u>			<u>**c**</u>			<u>**b**</u>	
PtrPGM1	-0.41	0.26	0.08	0.13	0.14	-0.26	0.64	0.66	0.73	-0.17	0.20	0.05	0.81	0.91	0.81	0.41	0.23	0.50
		<u>**a**</u>			<u>**b**</u>			<u>**d**</u>			<u>**ab**</u>			<u>**d**</u>			<u>**c**</u>	
PtrPGM2	-0.27	-0.44	-0.72	0.08	0.05	-0.12	0.92	0.90	0.86	-0.07	-0.20	-0.37	0.76	0.65	0.56	0.19	0.51	0.18
		<u>**a**</u>			<u>**b**</u>			<u>**c**</u>			<u>**b**</u>			<u>**d**</u>			<u>**b**</u>	
PtrPGM3	-0.56	-0.68	-0.49	0.05	0.00	-0.06	0.43	0.31	0.26	-0.01	0.09	0.02	0.67	0.66	0.65	0.16	-0.01	0.04
PtrPGM4		<u>**a**</u>			<u>**b**</u>			<u>**b**</u>			<u>**b**</u>			<u>**c**</u>			<u>**b**</u>	

	-2.10	-1.63	-0.81	-0.09	-0.06	0.15	0.21	0.01	0.08	-0.15	-0.21	-0.25	0.95	0.89	1.08	-0.18	-0.07	-0.11
		**a**			**a**			**d**			**a**			**c**			**b**	
PtrUGP1	0.11	0.13	-0.02	-0.05	0.03	0.02	0.92	0.94	0.97	0.12	0.05	-0.14	0.56	0.60	0.61	0.38	0.44	0.43
		**a**			**a**			**c**			**bc**			**c**			**b**	
PtrUGP2	-0.14	-0.01	-0.07	-0.19	0.16	0.02	0.27	0.39	0.44	0.15	0.28	0.21	0.36	0.31	0.33	0.16	0.14	0.17

The $2^{-\Delta\Delta CT}$ method was used to analyze the expression levels of genes. The values of log_2 (sample/control) upon to different nitrogen/carbon treatment conditions were calculated as the relative expression levels of every gene.1 mM NH_4NO_3 vs 400 ppm CO_2 was considered as the control. The statistical significant differences of the expression levels were analyzed using Duncan's multiple range test method ($P <0.05$). All the values under nitrogen/carbon treatment conditions were compared with the corresponding values under control condition.
