# Supplementary material for: Structure and Expression Analysis of PtrSUS, PtrINV, PtrHXK, PtrPGM, and PtrUGP Gene Families in Populus trichocarpa Torr. and Gray
Source: Int J Mol Sci. 2023 Dec 8;24(24):17277. doi: 10.3390/ijms242417277 (PMC10743687; doi:10.3390/ijms242417277)
Supplement: Supplementary file 1 [file ijms-24-17277-s001.zip › Table S7.pdf]

Table S7. The data of structural and non-structural carbon content, invertase activity, plant height, diameter, internode length, number of leaves and internodes in WT and KO-*PtrNINV12* lines.

Name (unit)	WT	KO-*PtrNINV12*-1	KO-*PtrNINV12*-2
**Starch (mg · g-1·Fw)**	7.531254	10.46946	9.997522
	7.181104	10.28678	10.13454
	8.368567	11.2611	11.21543
**Sucrose (mg · g-1·Fw)**	13.21232877	15.17123288	14.28082192
	13.17123288	14.59589041	15.65068493
	13.40410959	14.33561644	14.25342466
**Fructose (mg · g-1·Fw)**	6.879377	7.525292	7.354086
	6.762646	7.361868	7.361868
	6.988327	7.400778	7.089494
**Glucose (mg · g-1·Fw)**	1.543434	1.705051	1.60404
	1.573737	1.745455	1.644444
	1.513131	1.624242	1.725253
**Lignin (%)**	21.6261	20.48402	21.54453
	23.4208	21.6261	22.68661
	21.87084	22.52345	23.17607
	22.52345	23.09449	22.60503
**Cellulose (%)**	42.36364	39.27273	39.69697
	42.66667	38.24242	39.63636
	42	38.48485	40.18182
	42.42424	39.15152	40
	44	39.75758	37.93939
**Hemicellulos (%)**	18.42737	16.21684	16.13263
	17.54316	14.82737	17.08
	17.24842	15.43789	16.42737
**Height (cm)**	51	88	85
	55	86	86
	57	78	79
	65	89	82
	60	79	83
	64	85	80
	66	83	80
	60	86	84
	60	85	82
**Diameter (mm)**	4IN	4IN	4IN
	1.63	2.1	2.09
	1.55	2.19	2.18
	1.64	1.93	1.94
	2	1.95	1.94
	1.71	2	1.99

	1.73	1.98	1.98
	1.98	2.05	2.04
	2	2.06	2.05
		1.99	1.98
	8IN	8IN	8IN
	1.99	3.29	3.19
	2.29	2.8	2.79
	2.13	2.63	2.66
	2.09	2.83	2.82
	2.44	2.75	2.77
	2.34	3.3	3
	2.05	3.02	3.28
	2.44	2.99	2.88
		2.88	2.78
	16IN	16IN	16IN
	2.75	3.51	3.45
	2.78	3.38	3.28
	2.96	3.08	3.03
	3.01	3.45	3.25
	2.05	3.55	3.26
	2.7	3.46	3.36
		3.09	3.07
		3.12	3.11
		3.11	3.09
	GD	GD	GD
	2.87	4.16	3.99
	2.79	4.45	4.43
	3.11	3.55	3.56
	3.21	4.2	4.09
	3.29	4.03	4.13
	3.2	4.09	4.08
	2.73	4.13	4.11
	2.98	3.99	4.01
		3.89	4
**Invertase activity**	NINV	NINV	NINV
**(nmol/min/g)**	118.4014	111.2585	104.7959
	119.0816	113.9796	113.2993
	122.483	105.4762	114.3197
	CWINV	CWINV	CWINV
	346.6146	354.4271	314.8438
	325.2604	348.6979	360.1563
	317.9688	330.9896	362.2396
	VINV	VINV	VINV
	501.4286	510.9524	506.1905

	512.9932	535.4422	535.4422
	519.7959	516.3946	522.517
**Internode length (cm)**	4IN	4IN	4IN
	3.5	3.5	3.5
	4	4	3
	3.8	3.7	3.5
	3.5	3.5	3.9
	3	3.6	3.8
	3.5	3.5	3.5
	3.8	3.7	3.6
	3.8	3.5	3.7
		3.6	3.6
	8IN	8IN	8IN
	3.5	4	3.8
	3.5	3	3.2
	2	3.5	3.6
	3.5	3.6	3.5
	3.5	3.7	3.5
	3.8	3.6	3.5
	3	3.5	3.6
	3	3.5	3.5
		3.6	3.6
	16IN	16IN	16IN
	3.5	4	3.6
	3.5	4	3.5
	3.5	3	3.8
	3.5	3.8	3.7
	3.5	4.2	3.6
	4	3.8	3.8
	3	3.8	3.7
	3.5	3.5	3.5
		3.5	3.5
**Number of leaves and**	Leaf number	Leaf number	Leaf number
**Internode**	17	23	22
	16	22	23
	18	20	21
	20	21	20
	20	23	23
	19	20	20
	19	21	22
	18	22	21
		23	23
	Nodes number	Nodes number	Nodes number

	18	24	23
	17	23	21
	19	21	24
	20	22	21
	21	23	22
	20	21	23
	20	25	25
	19	24	21
		23	23
