# Supplementary material for: Structure and Expression Analysis of PtrSUS, PtrINV, PtrHXK, PtrPGM, and PtrUGP Gene Families in Populus trichocarpa Torr. and Gray
Source: Int J Mol Sci. 2023 Dec 8;24(24):17277. doi: 10.3390/ijms242417277 (PMC10743687; doi:10.3390/ijms242417277)
Supplement: Supplementary file 1 [file ijms-24-17277-s001.zip › Table S8.pdf]

Table S8. Primer sequences for qRT-PCR analysis.

Gene symbol	Locus name (POPTR)	Primers (5′-3′)	Product size (bp)	Annealing temp (°C)
PtrSUS1	Potri.018G063500.1	F: AAAACATTGGCAGGCGGTGC<br>R: GCACGAAGTTGCCGTTGCAG	220	60
PtrSUS2	Potri.006G136700.1	F: AAGTGCAAGGCTGATCCCAGT<br>R: AGATAGCGACGGCTCTCACGA	173	60
PtrSUS3	Potri.002G202300.1	F: TGCAGGACACTCTCTCCGCT<br>R: CGGCCACAAAAGGAGGCAGA	216	60
PtrSUS5	Potri.012G037200.1	F: GGCCAGTCAGATGTTCTGGGA<br>R: CTGGTTGCACTTTGTCCCTCCA	183	60
PtrSUS6	Potri.004G081300.1	F: TCTGTCAGAAAGCTTCGGATGGC<br>R: TGGATCCGGTGCTTGGAGAA	192	60
PtrSUS7	Potri.017G139100.1	F: ACAGGCGGACAGGTGGTCTA<br>R: TTGGTGCCATCAATTGCTTCG	179	60
PtrNINV1	Potri.008g101500.2	F: TTGATTCTGGGCTGTGGTGG<br>R: CCGAGTCTGATTCCTGTCTGG	106	60
PtrNINV2	Potri.013g006600.1	F: TTCAATGACCAGTGGGACGA<br>R: GGTGAGGTAATTCCAAGAAAACC	131	60
PtrNINV3	Potri.008g024100.1	F: AGGAAGAGGAGAGTTTGGCAAC<br>R: ACTACAGAATCCCGCAGTAGC	118	60
PtrNINV4	Potri.010g236100.1	F: TGACTGGTGAACACACTGACT<br>R: TCTCTACCTGTTCCTACCGCA	142	60
PtNINV5	Potri.005g010800.1	F: AGCTAAGTTGGTCAGTGCTGT<br>R: AGTTGCAGGAAAACCCTAGT	127	60
PtrNINV6	Potri.004g186500.1	F: AAGCAGCTATTGGCCGAGTT<br>R: TCCCAGTTTGCACATCGAC	123	60
PtrNINV7	Potri.005g239400.1	F: CTAGGAGCAGGAGTGATGCC<br>R: CTTCCTATCGCACTCTCGCC	101	60
PtNINV8	Potri.019g082000.1	F: TGGTGAGAGCGCAATTGGA<br>R: CCTTTCTGGCACTCTGGAGTTT	126	60
PtrNINV9	Potri.004g167500.1	F: GCTTGAAGAGTGTCGATGCC<br>R: TCACATGATCTTTGCCTCTCCA	115	60
PtrNINV10	Potri.002g173600.1	F: AATCGGAAGAGTGGCACCTG<br>R: TGTACCTCCGGGCGATCTAA	108	60
PtrNINV11	Potri.009g129000.1	F: TGCGATGAAAGGTCCCTAAGC<br>R: AACCCTGACCTTCTACCGGG	137	60
PtrNINV12	Potri.013g110800.1	F: TGCTCCAGTTGACTCTGGTT<br>R: CCCTTCTGACACTCTGGTCTC	102	60
PtrNINV13	Potri.014G100500.1	F: GGTGGCAGTGCAATAGGAAGA<br>R: CAAAGCCATCTGAGAGGCAG	112	60
PtrNINV14	Potri.014G188200.1	F: TCCTTGACTCTATCCGGGTGT<br>R: ATGGTAACTCCGCCGTGTG	149	60
PtrNINV15	Potri.014G188100.1	F: CAGAGAGTATTATTGGGTGGACA<br>R: CTAGCCATGAAGGAATTTGATCTTC	124	60
PtrNINV16	Potri.017G052800.1	F: AATCTCCCTGGTGACTCCCG<br>R: TTCCTTGCTTGCTCCCTTGG	119	60
PtrCWINV1	Potri.016g077400.1	F: CAAGGCAACCATGTTGAAGTT<br>R: GGAACCCTTTTGGGCACATAC	150	60

PtrCWINV2	Potri.016G077500.1	F: TTGGTCTGGTTCAGCGACAA<br>R: TTGCGGGTACTGCGTAGTTT	107	60
PtrCWINV3	Potri.006G210600.1	F: GGCGGGGCTCAGATATGATT<br>R: CAGACTCGTTAGCCCAACCC	101	60
PtrCWINV4	Potri.006g227500.1	F: GGGCCAATGTGGTATAAGGGG<br>R: GGCAAAGGGCATGATTGAGA	142	60
PtrCWINV5	Potri.006g227400.1	F: CAACGCAGCCTTCTGACATC<br>R: GTTCTTAGGCACTGCCAGGT	134	60
PtrVINV1	Potri.003g126300.1	F: AAGTTTTGCTCAAGGTGGTCG<br>R: AAGTGACGGTAGCCTCAATGG	116	60
PtrVINV2	Potri.003g112600.1	F: GCCACAATCCTCCCTGATGG<br>R: GGATCATCGTGGTCTGCTGG	104	60
PtrVINV3	Potri.015g127100.1	F: GTTCGGTTGTGCCACTTGAC<br>R: ATGGTCCCAAAGCACTCCTG	141	60
PtrHXK1	Potri.001G190400.1	F: TGTATGTGTGGCTGCGGTGG<br>R: GCTGCCACCTTCAGAGGCAA	205	60
PtrHXK2	Potri.001G254800.1	F: GGATTCCGAAAGCCCTAACCCAAA<br>R: TCTTAGCAACTTCCTTCAGCTCAG	221	60
PtrHXK3	Potri.005G238600.1	F: CTGCCGCATGGAAGGCAAAG<br>R: TAACCAGGGCAGACACACGC	196	60
PtrHXK4	Potri.007G009300.1	F: AAGGGGATGAGAAGGGGCTGT<br>R: TGGGGGATGCTCTGAAACGA	212	60
PtrHXK5	Potri.009G050000.1	F: GCTGGCTGCAATGCATGAGG<br>R: TCCACTTCCATCCCGTCCGA	205	60
PtrHXK6	Potri.018G088300.1	F: CAGTAGGAGCGGCGGTTGTT<br>R: AAGGCCGGCATGCATCTCAA	197	60
PtrPGM1	Potri.008G132500.3	F: GGCATGCTTGGTCAAACTGCAA<br>R: ACCAGTTCCTGAGAGGCGGA	202	60
PtrPGM2	Potri.010G109500.2	F: AGGAGCATGCCAACTTCTTCTGG<br>R: AGCCAGAACTGCCCAAATTCCA	195	60
PtrPGM3	Potri.012G132500.1	F: TATGCAGAGAACGGGCCAGA<br>R: CTTGTTGGCATGGACCGAGC	182	60
PtrPGM4	Potri.015G134700.1	F: TTGGGGCCAGTCCGGATTCA<br>R: CTTCCTGTGCATTGGCAGCA	237	60
PtrUGP1	Potri.004G074400.5	F: GGTTGCCAACTTCTTGAGCCG<br>R: TCAAGAACGACTCCCTCTGGT	171	60
PtrUGP2	Potri.017G144700.1	F: GCTTGATAGTCTAAAGGTGGTCGGT<br>R: TAGAGGTCCTCCGGGCCATT	156	60
PtrUBQ7	Potri.0005G22060.1	F: GGAACGGGTTGAGGAGAAAGAAG<br>R: GCAAGAACAAGATGAAGCACAGAGC	135	60
