# Supplementary material for: Structure and Expression Analysis of PtrSUS, PtrINV, PtrHXK, PtrPGM, and PtrUGP Gene Families in Populus trichocarpa Torr. and Gray
Source: Int J Mol Sci. 2023 Dec 8;24(24):17277. doi: 10.3390/ijms242417277 (PMC10743687; doi:10.3390/ijms242417277)
Supplement: Supplementary file 1 [file ijms-24-17277-s001.zip › Table S9.pdf]

Table S9. Primer sequences of vector.

Primer name	Primers (5′-3′)
Construct 2gRNA primers	
NINV12-DT1-BsF	ATATATGGTCTCGATTGCTCTAAGCGAGCTCTCGATGTT
NINV12-DT1-F0	TGCTCTAAGCGAGCTCTCGATGTTTTAGAGCTAGAAATAGC
NINV12-DT2-R0	AACTTGAGACCACATATTCACCCAATCTCTTAGTCGACTCTAC
NINV12-DT2-BsR	ATTATTGGTCTCGAAACTTGAGACCACATATTCACCCAA
Hi-TOM platform sequencing primer	
NINV12/DT1/2 Cas9-F	GGAGTGAGTACGGTGTGCAGGCAGAGATCGTTTGATGAGAG
NINV12/DT1/2Cas9-R	GAGTTGGATGCTGGATGGCCTCTGAGGCATGGTCATATGCTG
